# Supplementary material for: Prevalence of vitamin D and calcium deficiencies and their health impacts on women of childbearing age: a protocol for systematic review and meta-analysis
Source: BMJ Open. 2022 May 4;12(5):e049731. doi: 10.1136/bmjopen-2021-049731 (PMC9083400; doi:10.1136/bmjopen-2021-049731)
Supplement: Supplementary data [file bmjopen-2021-049731supp001.pdf]

Supplementary Table. Search terms selected according to specific nomenclature for each database

**MEDLINE**	(("Micronutrients" [Title/Abstract] OR "Micronutrients" [MeSH Terms] OR "Deficiency Diseases" [Title/Abstract] OR "Deficiency Diseases" [MeSH Terms] OR "Avitaminosis" [Title/Abstract] OR "Avitaminosis" [MeSH Terms] OR "Deficiencies vitamin" [Title/Abstract] OR "Deficiencies vitamin" [MeSH Terms] OR "Deficiency vitamin" [Title/Abstract] OR "Vitamin deficiency" [Title/Abstract] OR "Vitamin Deficiencies" [Title/Abstract] OR "Serum Levels" [Title/Abstract] OR "Nutritional Status" [Title/Abstract] OR "Nutritional Status" [MeSH Terms] OR "Reduced Level" [Title/Abstract] OR "Reduced Level" [MeSH Terms]) AND ("Women" [Title/Abstract] OR "Women" [MeSH Terms] OR "Girl" [Title/Abstract] OR "Girls" [Title/Abstract] OR "Woman" [Title/Abstract] OR "Adolescent" [Title/Abstract] OR "Adolescent" [MeSH Terms] OR "Teen" [Title/Abstract] OR "Teens" [Title/Abstract] OR "Teenager" [Title/Abstract] OR "Pregnancy" [Title/Abstract] OR "Pregnancy" [MeSH Terms] OR "Gestation" [Title/Abstract] OR "Pregnancies" [Title/Abstract])) AND ("Vitamin D" [Title/Abstract] OR "Vitamin D" [MeSH Term] OR "Vitamin D Deficiency" [Title/Abstract] OR "Vitamin D Deficiency" [MeSH Term] OR "deficiencies vitamin d" [Title/Abstract] OR "Vitamin D Deficiencies" [Title/Abstract] OR "Vitamin D Deficiencies" [MeSH Term] OR "Calcium" [Title/Abstract] OR "Calcium" [MeSH Term] OR "Hypocalcemia" [Title/Abstract] OR "Hypocalcemia" [MeSH Term] OR "Hypocalcemias" [Title/Abstract])
**Embase**	('micronutrients':ab,ti OR 'deficiency diseases':ab,ti OR 'avitaminosis':ab,ti OR 'avitaminoses':ab,ti OR 'deficiencies vitamin':ab,ti OR 'deficiency vitamin':ab,ti OR 'vitamin deficiency':ab,ti OR 'vitamin deficiencies':ab,ti OR 'serum levels':ab,ti OR 'nutritional status':ab,ti OR 'reduced level':ab,ti) AND ('women':ab,ti OR 'girl':ab,ti OR 'girls':ab,ti OR 'woman':ab,ti OR 'adolescent':ab,ti OR 'teen':ab,ti OR 'teens':ab,ti OR 'teenager':ab,ti OR 'pregnancy':ab,ti OR 'gestation':ab,ti OR 'pregnancies':ab,ti) AND ('vitamin d':ab,ti OR 'vitamin d deficiency':ab,ti OR 'deficiencies vitamin d':ab,ti OR 'vitamin d deficiencies':ab,ti OR 'calcium':ab,ti OR 'hypocalcemia':ab,ti OR 'hypocalcemias':ab,ti)
**Scopus**	(TITLE-ABS ("Micronutrients" OR "Deficiency Diseases" OR "Avitaminosis" OR "Avitaminoses" OR "Deficiencies, Vitamin" OR "Deficiency Vitamin" OR "Vitamin Deficiency" OR "Vitamin Deficiencies" OR "Serum Levels" OR "Nutritional Status" OR "Reduced Level")) AND (TITLE-ABS ("Women" OR "Girl" OR "Girls" OR "Woman" OR "Adolescent" OR "Teen" OR "Teens" OR "Teenager" OR "Pregnancy" OR "Gestation" OR "Pregnancies")) AND (TITLE-ABS ("Vitamin D" OR "Vitamin D Deficiency" OR "Deficiencies, Vitamin D" OR "Vitamin D Deficiencies" OR "Calcium" OR "Hypocalcemia" OR "Hypocalcemias"))
**Web of Science**	(TI= "Micronutrients" OR AB= "Micronutrients" OR TI="Deficiency Diseases" OR AB= "Deficiency Diseases" OR TI= "Avitaminosis" OR AB= "Avitaminosis" OR TI= "Avitaminoses" OR AB= "Avitaminoses" OR TI= "deficiencies vitamin" OR AB= "deficiencies vitamin" OR TI= "Deficiency Vitamin" OR AB= "Deficiency Vitamin" OR TI= "Vitamin Deficiency" OR AB="Vitamin Deficiency" OR TI= "Vitamin Deficiencies" OR AB="Vitamin Deficiencies" OR TI= "Serum Levels" OR AB= "Serum Levels" OR TI="Nutritional Status" OR AB="Nutritional Status" OR TI="Reduced Level" OR AB="Reduced Level") AND (TI="Women" OR AB= "Women" OR TI="Girl" OR AB="Girl" OR TI="Girls" OR AB="Girls"

	OR TI="Woman" OR AB="Woman" OR TI="Adolescent" OR AB= "Adolescent" OR TI="Teen" OR AB="Teen" OR TI="Teens" OR AB="Teens" OR TI="Teenager" OR AB="Teenager" OR TI="Pregnancy" OR AB="Pregnancy" OR TI="Gestation" OR AB="Gestation" OR TI="Pregnancies" OR AB="Pregnancies") AND (TI="Vitamin D" OR AB="Vitamin D" OR TI="Vitamin D Deficiency" OR AB= "Vitamin D Deficiency" OR TI="deficiencies vitamin d" OR AB="deficiencies vitamin d" OR TI="Vitamin D Deficiencies" OR AB="Vitamin D Deficiencies" OR TI="Calcium" OR AB="Calcium" OR TI="Hypocalcemia" OR AB= "Hypocalcemia" OR TI="Hypocalcemias" OR AB= "Hypocalcemias")
**LILACS**	(tw:((tw:("Micronutrients")) OR (tw:("Deficiency Diseases")) OR (tw:("Avitaminosis")) OR (tw:("Avitaminoses")) OR (tw:("deficiencies vitamin")) OR (tw:("Deficiency Vitamin")) OR (tw:("Vitamin Deficiency")) OR (tw:("Vitamin Deficiencies")) OR (tw:("Serum Levels")) OR (tw:("Nutritional Status")) OR (tw:("Reduced Level")))) AND (tw:(Women OR Girl OR Girls OR Woman OR Adolescent OR Teen OR Teens OR Teenager OR Pregnancy OR Gestation OR Pregnancies)) AND (tw:(("Vitamin D") OR ("Vitamin D Deficiency") OR ("deficiencies vitamin d") OR ("Vitamin D Deficiencies") OR "Calcium" OR "Hypocalcemia" OR "Hypocalcemias"))
