# Supplementary material for: Prevalence of vitamin D and calcium deficiencies and their health impacts on women of childbearing age: a protocol for systematic review and meta-analysis
Source: BMJ Open. 2022 May 4;12(5):e049731. doi: 10.1136/bmjopen-2021-049731 (PMC9083400; doi:10.1136/bmjopen-2021-049731)
Supplement: Author's manuscript [file bmjopen-2021-049731.draft_revisions.pdf]

BMJ Open is committed to open peer review. As part of this commitment we make the peer review history of every article we publish publicly available.

When an article is published we post the peer reviewers' comments and the authors' responses online. We also post the versions of the paper that were used during peer review. These are the versions that the peer review comments apply to.

The versions of the paper that follow are the versions that were submitted during the peer review process. They are not the versions of record or the final published versions. They should not be cited or distributed as the published version of this manuscript.

BMJ Open is an open access journal and the full, final, typeset and author-corrected version of record of the manuscript is available on our site with no access controls, subscription charges or pay-per-view fees (http://bmjopen.bmj.com).

If you have any questions on BMJ Open's open peer review process please email info.bmjopen@bmj.com

BMJ Open

Prevalence of vitamin D and calcium deficiencies and their health impacts on women of childbearing age: a protocol for systematic review and meta-analysis

Journal:	*BMJ Open*
Manuscript ID	bmjopen-2021-049731
Article Type:	Protocol
Date Submitted by the Author:	01-Feb-2021

Complete List of Authors:	Silveira, Erika; Federal University of Goias, Department of Clinical Medicine; University College London, Department of Clinical Medicine<br>Nogueira, Letícia; Federal University of Goias, Department of Clinical Medicine<br>Castro, Maria ; Federal University of Goias, Department of Clinical Medicine<br>Kac, Gilberto; Federal University of Rio de Janeiro<br>Noll, Priscilla; Goiano Federal Institute, Department of Obstetrics and Gynecology; University of São Paulo, Department of Obstetrics and Gynecology<br>de Oliveira, Cesar; University College London, Epidemiology and Public Health<br>Noll, Matias; Instituto Federal Goiano, Public Health; University of Southern Denmark,

Keywords:	NUTRITION & DIETETICS, EPIDEMIOLOGY, Protocols & guidelines < HEALTH SERVICES ADMINISTRATION & MANAGEMENT

SCHOLARONE™
Manuscripts

Prevalence of vitamin D and calcium deficiencies and their health impacts on women of childbearing age: a protocol for systematic review and meta-analysis

Erika Aparecida Silveira[1,2]

Letícia de Almeida Nogueira e Moura[1]

Maria Clara Rezende Castro[1]

Gilberto Kac[3]

Priscilla Noll[4,5]

Cesar de Oliveira[2]

Matias Noll[4,6]

[1]Department of Clinical Medicine, Faculty of Medicine, Health Science Post-graduate Program, Federal University of Goias, Goiás, Brazil

[2]Department of Epidemiology & Public Health, Institute of Epidemiology & Health Care, University College London, London, UK

[3]Federal University of Rio de Janeiro, Rio de Janeiro, Brazil

[4]Goiano Federal Institute, Goiás, Brazil

[5]Department of Obstetrics and Gynecology, Medical School, University of São Paulo, São Paulo, Brazil

[6]Department of Sports Science and Clinical Biomechanics, University of Southern Denmark, Odense, Denmark

Correspondentauthor: Erika A.Silveira:erikasil@terra.com.br

For peer review only

ABSTRACT

Introduction: Previous studies have investigated the prevalence of vitamin D and calcium deficiencies in women of childbearing age. However, there are no systematic reviews that synthesize data on the available evidence to determine the extent of the prevalence of calcium and vitamin D deficiencies as a public health problem globally. Therefore, this study presents a protocol for conducting a review and meta-analysis to estimate the prevalence of calcium and vitamin D deficiencies in women of childbearing age and stratify these data by age group, urban and rural area, world region, and pregnant/non-pregnant women. **Methods and analysis:** The systematic review protocol involves conducting a literature search in the following databases: PubMed, LILACS, Embase, Scopus and Web of Science. The population under investigation will be women of childbearing age. The selected articles will be checked thoroughly, including the references to include gray literature. Cross-sectional studies and baseline data from cohort studies or clinical and community trials conducted with women of childbearing age with representative probabilistic sampling will be included. Two independent researchers will be responsible for article selection and data extraction, and discrepancies, if any, will be dealt with by a third reviewer. Methodological quality and risk of bias will be analyzed using the Grading of Recommendations, Assessment, Development, and Evaluations, and Downs and Black scales, respectively. The heterogeneity of the estimates between studies will also be evaluated. The results will be presented in a report format that will systematically organize evidence on the prevalence of the most critical micronutrient deficiencies in women of childbearing age, emphasizing calcium and vitamin D levels in pregnant and non-pregnant women. Dissemination of the key findings from the systematic review will help to identify priorities for action, establishing dietary guidelines, developing health-related public policies, and reducing and combating micronutrient deficiencies among women of childbearing age and their children.

Ethics and dissemination: The protocol was written according to the Preferred Reporting Items for Systematic Reviews and Meta-Analyses. No ethical approval is required.

Systematic review registration: PROSPERO CRD42020207850.

Keywords: Micronutrients, Calcium, Vitamin D, Childbearing age, Systematic Review Protocol, Nutritional Deficiencies, Epidemiology

For peer review only

Strengths and limitations of this study

►This is the first systematic reviews aiming to estimate the prevalence of calcium and vitamin D deficiencies in women of childbearing age and stratify these data by age group, urban and rural area, world region, and pregnant/non-pregnant women;

► An extensive literature search will be performed using six databases with a peer-reviewed search strategy according to the scoping review guidelines;

► Dissemination of this study and subsequent findings from this systematic review may contribute to the identification of priorities for action, establishing dietary guidelines, developing health-related public policies, and reducing and combating micronutrient deficiencies among women of childbearing age and their children.

For peer review only

INTRODUCTION

Nutritional deficiencies have multiple etiologies related to factors such as nutrient loss, malabsorption, inadequate intake, and even ignorance of the importance of nutritional care during pregnancy[1]. Women of childbearing age are part of the highly vulnerable population and often experience these deficiencies, especially during pregnancy[2–5]. Besides, maternal nutritional status influences the pregnancy outcomes and child's health and, as it is necessary to supply the needs of both the mother and the fetus[3–5].

Vitamin D deficiency is considered a significant public health problem and affects approximately one billion people worldwide[6,7]. However, there are significant data gaps in measuring this problem magnitude in many countries[8]. The estimated prevalence of vitamin D deficiency (20ng/ml) was around 37% in Canada and 40% in Europe[9]. It has been associated with adverse events such as an increased risk of cardiovascular disease, infection, cancer, bone loss, fractures, and mortality[9–13]. During the gestational period, on the one hand, this condition predisposes the mother to develop gestational diabetes, pre-eclampsia, and postpartum depression [8,14–17]. On the other hand, the fetus has an increased risk of premature birth and intrauterine growth restriction, low birthweight, in addition to a higher probability of developing allergies and obesity in the long term[7,18,19].

Vitamin D levels interfere with calcium metabolism [20]. Calcium is a micronutrient that plays an essential role in muscle contraction, in addition to regulating water balance in cells[21]. Hypocalcemia symptoms, characterized by low calcium levels, are variable ,ranging from mild disorders such as perioral paresthesia to severe life-threatening ones such as cardiac arrhythmias [22]. In pregnant women, this imbalance is characterized by a lower serum concentration and a higher concentration in the cells, thereby causing increased blood pressure in women with pre-eclampsia[23,24].

Previous studies have evaluated the prevalence of vitamin D and calcium deficiencies in women of childbearing age[25–27]. However, at present, there is no systematic review that synthesizes the panorama of this relevant public health problem nationally and globally. Therefore, this study aimed to estimate the prevalence of calcium and vitamin D deficiencies in women of childbearing age. In addition, the data will be

For peer review only

stratify by age group, urban and rural areas, geographical location, and pregnant/non-pregnant women through a systematic review followed by a meta-analysis.

METHODS AND ANALYSIS

This systematic review and meta-analysis will be written according to the Preferred Reporting Items for Systematic Reviews and Meta-Analyses Protocols (PRISMA). The protocol for this systematic review was registered on the Prospero platform (CRD42020207850). This review follows the Population, Intervention, Comparison, and Outcome (PICO) structure, with the population (P) women of childbearing age, the intervention (I) no intervention on this study, the comparison (C) being conducted between sub groups in childbearing age as pregnant and not pregnant, and the outcome (O) being calcium and vitamin D deficiencies.

Search strategy and databases

A systematic review of the literature will be conducted by identifying and collecting bibliographic data in the following databases: PubMed (National Library of Medicine, United States), LILACS (Latin American and Caribbean Health Sciences Literature), Embase, Scopus, and Web of Science databases (Figure 1). The search strategy will be complemented by screening the reference list of the included studies and relevant systematic reviews.

Different search strategies will include Medical Subject Heading (MeSH) terms and relevant keywords related to vitamin D deficiency and specific terms related to calcium deficiency to ensure that all articles of interest are identified using all the descriptors and Boolean operators of this systematic review protocol (**Table1**).

For peer review only

Table 1. Search terms selected according to specific nomenclature for each database

**MEDLINE**	(("Micronutrients" [Title/Abstract] OR "Micronutrients" [MeSH Terms] OR "Deficiency Diseases" [Title/Abstract] OR "Deficiency Diseases" [MeSH Terms] OR "Avitaminosis" [Title/Abstract] OR "Avitaminosis" [MeSH Terms] OR "Deficiencies vitamin" [Title/Abstract] OR "Deficiencies vitamin" [MeSH Terms] OR "Deficiency vitamin" [Title/Abstract] OR "Vitamin deficiency" [Title/Abstract] OR "Vitamin Deficiencies" [Title/Abstract] OR "Serum Levels" [Title/Abstract] OR "Nutritional Status" [Title/Abstract] OR "Nutritional Status" [MeSH Terms] OR "Reduced Level" [Title/Abstract] OR "Reduced Level" [MeSH Terms]) AND ("Women" [Title/Abstract] OR "Women" [MeSH Terms] OR "Girl" [Title/Abstract] OR "Girls" [Title/Abstract] OR "Woman" [Title/Abstract] OR "Adolescent" [Title/Abstract] OR "Adolescent" [MeSH Terms] OR "Teen" [Title/Abstract] OR "Teens" [Title/Abstract] OR "Teenager" [Title/Abstract] OR "Pregnancy" [Title/Abstract] OR "Pregnancy" [MeSH Terms] OR "Gestation" [Title/Abstract] OR "Pregnancies" [Title/Abstract])) AND ("Vitamin D" [Title/Abstract] OR "Vitamin D" [MeSH Term] OR "Vitamin D Deficiency" [Title/Abstract] OR "Vitamin D Deficiency" [MeSH Term] OR "deficiencies vitamin d" [Title/Abstract] OR "Vitamin D Deficiencies" [Title/Abstract] OR "Vitamin D Deficiencies" [MeSH Term] OR "Calcium" [Title/Abstract] OR "Calcium" [MeSH Term] OR "Hypocalcemia" [Title/Abstract] OR "Hypocalcemia" [MeSH Term] OR "Hypocalcemias" [Title/Abstract])
**Embase**	('micronutrients':ab,ti OR 'deficiency diseases':ab,ti OR 'avitaminosis':ab,ti OR 'avitaminoses':ab,ti OR 'deficiencies vitamin':ab,ti OR 'deficiency vitamin':ab,ti OR 'vitamin deficiency':ab,ti OR 'vitamin deficiencies':ab,ti OR 'serum levels':ab,ti OR 'nutritional status':ab,ti OR 'reduced level':ab,ti) AND ('women':ab,ti OR 'girl':ab,ti OR 'girls':ab,ti OR 'woman':ab,ti OR 'adolescent':ab,ti OR 'teen':ab,ti OR 'teens':ab,ti OR 'teenager':ab,ti OR 'pregnancy':ab,ti OR 'gestation':ab,ti OR 'pregnancies':ab,ti) AND ('vitamin d':ab,ti OR 'vitamin d deficiency':ab,ti OR 'deficiencies vitamin d':ab,ti OR 'vitamin d deficiencies':ab,ti OR 'calcium':ab,ti OR 'hypocalcemia':ab,ti OR 'hypocalcemias':ab,ti)
**Scopus**	(TITLE-ABS ("Micronutrients" OR "Deficiency Diseases" OR "Avitaminosis" OR "Avitaminoses" OR "Deficiencies, Vitamin" OR "Deficiency Vitamin" OR "Vitamin Deficiency" OR "Vitamin Deficiencies" OR "Serum Levels" OR "Nutritional Status" OR "Reduced Level")) AND (TITLE-ABS ("Women" OR "Girl" OR "Girls" OR "Woman" OR "Adolescent" OR "Teen" OR "Teens" OR "Teenager" OR "Pregnancy" OR "Gestation" OR "Pregnancies")) AND (TITLE-ABS ("Vitamin D" OR "Vitamin D Deficiency" OR "Deficiencies, Vitamin D" OR "Vitamin D Deficiencies" OR "Calcium" OR "Hypocalcemia" OR "Hypocalcemias"))
**Web of Science**	(TI= "Micronutrients" OR AB= "Micronutrients" OR TI="Deficiency Diseases" OR AB= "Deficiency Diseases" OR TI= "Avitaminosis" OR AB= "Avitaminosis" OR TI= "Avitaminoses" OR AB= "Avitaminoses" OR TI= "deficiencies vitamin" OR AB= "deficiencies vitamin" OR TI= "Deficiency Vitamin" OR AB= "Deficiency Vitamin" OR TI= "Vitamin Deficiency" OR AB="Vitamin Deficiency" OR TI= "Vitamin Deficiencies" OR AB="Vitamin Deficiencies" OR TI= "Serum Levels" OR AB= "Serum Levels" OR TI="Nutritional Status" OR AB="Nutritional Status" OR TI="Reduced Level" OR AB="Reduced Level") AND (TI="Women" OR AB= "Women" OR TI="Girl" OR AB="Girl" OR TI="Girls" OR AB="Girls" OR TI="Woman" OR AB="Woman" OR TI="Adolescent" OR AB= "Adolescent" OR TI="Teen" OR AB="Teen" OR TI="Teens" OR AB="Teens" OR TI="Teenager" OR

For peer review only

	AB="Teenager" OR TI="Pregnancy" OR AB="Pregnancy" OR TI="Gestation" OR AB="Gestation" OR TI="Pregnancies" OR AB="Pregnancies") AND (TI="Vitamin D" OR AB="Vitamin D" OR TI="Vitamin D Deficiency" OR AB= "Vitamin D Deficiency" OR TI="deficiencies vitamin d" OR AB="deficiencies vitamin d" OR TI="Vitamin D Deficiencies" OR AB="Vitamin D Deficiencies" OR TI="Calcium" OR AB="Calcium" OR TI="Hypocalcemia" OR AB= "Hypocalcemia" OR TI="Hypocalcemias" OR AB= "Hypocalcemias")
**LILACS**	(tw:((tw:("Micronutrients")) OR (tw:("Deficiency Diseases")) OR (tw:("Avitaminosis")) OR (tw:("Avitaminoses")) OR (tw:("deficiencies vitamin")) OR (tw:("Deficiency Vitamin")) OR (tw:("Vitamin Deficiency")) OR (tw:("Vitamin Deficiencies")) OR (tw:("Serum Levels")) OR (tw:("Nutritional Status")) OR (tw:("Reduced Level")))) AND (tw:(Women OR Girl OR Girls OR Woman OR Adolescent OR Teen OR Teens OR Teenager OR Pregnancy OR Gestation OR Pregnancies)) AND (tw:(("Vitamin D") OR ("Vitamin D Deficiency") OR ("deficiencies vitamin d") OR ("Vitamin D Deficiencies") OR "Calcium" OR "Hypocalcemia" OR "Hypocalcemias"))

Eligibility criteria

Inclusion criteria

- Studies that provide information on the prevalence of calcium and/or vitamin D deficiency in women of childbearing age (15-49 years old or menarche and menopause).
- Studies with representative population-based samples, in hospitals, health centers, or outpatient clinics.
- Prevalence data on women of different age groups, such as adolescents, pregnant women, lactating women, and premenopausal adult women.
- Studies with a cross-sectional design, longitudinal study baseline data (cohort studies), or analysis data from some point in the cohort study time provided that characterizes prevalence and baseline data from intervention studies such as clinical trials or community trials.
- No publication time or language limits will be imposed.

Exclusion criteria

- Opinion articles, comments, or editorials.
- Duplicates: for studies published as more than one article, the most comprehensive one that reports the largest sample size will be considered.
- Studies with primary data not accessible even after request.

For peer review only

- Case-control studies, narrative reviews, and case series.
- Studies conducted among athlete women of any sports modalities.
- Studies conducted among women with specific diseases (HIV/AIDS, dengue fever, cancer, kidney failure, and heart failure).
- Studies conducted among indigenous women.

Reviewers' training

The authors responsible for assessing article eligibility will be trained on using the inclusion and exclusion criteria. Thus, an eligibility test will be conducted on 50 titles and abstracts before codifying the articles. They will also receive training on instruments to assess the risk of bias, which will be used on five articles that have not been included. Rayyan and Mendeley software will be used for the selection steps.

Review process

After completing the search strategy, the identified articles will be gathered and imported into Mendeley software. Duplicate articles will be deleted. The articles will be selected by two independent reviewers (LM and MC). In the first stage, the titles will be read, and in the second stage, the abstracts will be read. Finally, in the third stage, the full article will be read. Disagreements between reviewers will be assessed by a third reviewer (EAS). Eligibility was determined according to the inclusion and exclusion criteria. The percentage of agreement and the Kappa coefficient will be calculated to assess reliability between reviewers' assessment. At the end of the process, the selected articles will be included in the systematic review.

Data extraction and risk of bias assessment

Data extraction will be performed using a spreadsheet that considers the following aspects: author/year, type of study, place of study, age group, type of sample/sample size, place of residence (urban/rural), continent/ world region (Asia, Europe, Africa, Latin America, North America, and Oceania), apregnant woman (yes/no), lactating (yes/no), micronutrients analyzed, the technique used, cutoff points, and results i.e. prevalence/impact of calcium or vitamin D deficiencies (Table 2).

For peer review only

The Downs and Black tool [28] will be used to analyze the quality and risk of bias and its adaptation for observational studies[29]. Considering the inclusion of observational studies, some items on the Downs and Black checklist will not be applicable. Thus, 19 questions will be selected, and a score of 0-19 points will be assigned to each article. The low risk of bias will be determined when the score is above 70%.

The Grading of Recommendations, Assessment, Development and Evaluations (GRADE)[30] will be used to assess the strength of evidence. The quality of evidence will be classified into four grades: high, moderate, low, or very low.

The data will be extracted and evaluated by two independent reviewers (LM and MC). Disagreements will be resolved by a third reviewer (MN). To obtain relevant data that are not described in the manuscript, a researcher (MC) will contact the article's authors. The authors of the included studies' potential conflicts of interest and ethical information will also be described.

Statistical analysis

The possibility of performing a meta-analysis will be evaluated using fixed-effects or random-effects methods according to the studies' homogeneity. Chi-square test with a significance level ($p < 0.05$) will be used to assess heterogeneity, and the I-square statistic (I^2) will be used to assess the magnitude of inconsistency. I^2 results above 75% will indicate ahigh heterogeneity, those between 25% and75% will indicate moderate heterogeneity, and those below 25% will indicate low heterogeneity[31–33].

According to the available literature, sensitivity and estimation of the prevalence of vitamin D and calcium deficiencies will be analyzed, and the data will be stratified according to region, age, and urban/rural area subgroups. If applicable, meta-regression will be performed to explain the studies' heterogeneity considering $p < 0.05$ for each stratification subgroup.

The main outcome of the present study is the prevalence of serum calcium and vitamin D deficiencies in women of childbearing age. A 95% confidence interval for prevalence will be considered.

DISCUSSION

For peer review only

Nutritional deficiencies affect pregnant and non-pregnant women, especially those living in poorer regions such as the Brazilian northeast [34–38]. Thus, such nutritional deficiencies need to be reviewed as public health problems[39,40], nationally and globally. Therefore, it is important to estimate the extension of this problem. To the best of our knowledge, there are no systematic reviews and meta-analyses that synthesize data on the available evidence to assess the extent of the prevalence of calcium and vitamin D deficiencies in women of childbearing age.

Obstacles in implementing policies and public health programs aimed at combating nutritional deficiencies can be exacerbated by inequalities in health service provision and access[41]. Therefore, it is essential to gather, organize, and systematize the available data on the prevalence of the main vitamin and mineral deficiencies for developing intervention plans, listing action priorities, and improving existing public health policies for women of childbearing age.

This systematic review protocol is important to improve knowledge on global and Brazilian realities on the prevalence of vitamin D and calcium deficiency in this population group. This study is expected to contribute to suggestions for priority action strategies and the development of public health policies to minimize the problem and prevent the occurrence of new cases and assist in the establishment of intervention protocols to minimize the main deficiencies according to the priorities indicated in the final report. However, some limitations of this research need to be highlighted. First, the potential heterogeneity of measurements and assessments of micronutrient deficiency in research. Second, studies may have used different methods making it difficult to conduct a meta-analysis.

Women of childbearing age, especially pregnant women, are part of a highly vulnerable group that develops nutritional deficiencies, and the maternal nutritional status influences gestational outcomes and the child's health. Considering the above, assessing the prevalence of vitamin D and calcium deficiencies could help identifying knowledge gaps on the magnitude of these deficiencies, warranting further studies. From this perspective, disseminating the results of this systematic review protocol will be useful and important to encourage research and identifying priorities, establishing dietary guidelines, and reducing and combating micronutrient deficiencies in this population.

For peer review only - http://bmjopen.bmj.com/site/about/guidelines.xhtml

Patient and public involvement

No patient involved.

Ethics and Dissemination

A systematic review does not require ethical approval since it synthesizes publicly available literature. The results of the scoping review will be submitted for publication to a scientific journal and presented at relevant conferences

Acknowledgment

The authors thank CNPq and IF Goiano for providing partial support.

Funding sources/sponsors:

This study received support from the This study received support from the MS-SCTIE-Decit/*CNPq* Nº 442932/2019-7. Dr Cesar de Oliveira is supported by the Economic and Social Research Council (Grant ES/T008822/1)

Conflicts of interest:

The authors of this systematic review and meta-analysis declare no conflicts of interest. This review will analyze whether the studies report a possible conflict of interest and approval by the Research Ethics Committee (ELIA et al., 2016). It will also verify if the studies followed the ethical requirements recommended in the Declaration of Helsinki, respecting all the ethical principles established in resolution 466/2012 of the National Health Council (BRASIL, 2012) or the norms and ethical resolutions in force at the time of data collection.

REFERENCES

1. Miranda VIA, Dal Pizzol TS, Silveira MPT, Mengur SS, Da Silveira MF, Luz, et al. The use of folic acid, iron salt sand other vitamins by pregnant women in the 2015 Pelotas birth cohort: is there socioeconomic inequality? BMC Public Health. 2019;19:1–8.

For peer review only - http://bmjopen.bmj.com/site/about/guidelines.xhtml

2. Barger MK. Maternal nutrition and perinatal outcomes. J Midwifery Women's Health. 2010;55:502–11.

3. Iqbal S, Ekmekcioglu C. Maternal and neonatal outcomes related to iron supplementation or iron status: a summaryof meta-analyses. The Journalof Maternal-Fetal & Neonatal Medicine [Internet]. 2019;32:1528–40. Available from: https://www.tandfonline.com/doi/full/10.1080/14767058.2017.1406915

4. Obeid R, Murphy M, Solé-Navais P, Yajnik C. Cobalamin Status from Pregnancy to Early Childhood: Lessons from Global Experience. Advances in Nutrition: An International Review Journal [Internet]. 2017;8:971–9. Available from: http://advances.nutrition.org/lookup/doi/10.3945/an.117.015628

5. Nauman N, Jalali S, Shami S, Rafiq S, Große G, Hilger AC, et al. Low maternal folate concentrations and maternal MTHFR C677T polymorphism are associated with an increase drisk for neural tube defects in offspring: A case-control study among Pakistani case and control mothers. Asia Pacific Journal of Clinical Nutrition. HEC Press; 2018;27:253–60.

6. Mithal A, Wahl DA, Bonjour J-P, Burckhardt P, Dawson-Hughes B, Eisman JA, et al. Global vitamin D status and determinants of hypovitaminosis D. Osteoporosis International [Internet]. 2009;20:1807–20. Available from: http://link.springer.com/10.1007/s00198-009-0954-6

7. Martins MEP, Esmeraldo CUP, Sabiá JPD, Carvalho JHL, Suano-Souza FI, Sarni ROS. Vitamin D Post partum Concentrations: Relationship with Nutritional Condition and Morbidities during Pregnancy. Journal of Pregnancy [Internet]. 2018;2018:1–6. Available from: https://www.hindawi.com/journals/jp/2018/1070528/

8. Roth DE, Abrams SA, Aloia J, Bergeron G, Bourassa MW, Brown KH, et al. Global prevalence and disease burden of vitamin D deficiency: a road map for action in low and middle-income countries. Annals of the New York Academy of Sciences [Internet]. 2018;1430:44–79. Available from: https://onlinelibrary.wiley.com/doi/abs/10.1111/nyas.13968

9. Amrein K, Scherkl M, Hoffmann M, Neuwersch-Sommeregger S, Köstenberger M, TmavaBerisha A, et al. Vitamin D deficiency 2.0: an update on the current status worldwide. European Journal of Clinical Nutrition [Internet]. 2020;74:1498–513. Available from: http://www.nature.com/articles/s41430-020-0558-y

10. Garland CF, Kim JJ, Mohr SB, Gorham ED, Grant WB, Giovannucci EL, et al. Meta-analysis of All-Cause Mortality According to Serum 25-Hydroxyvitamin D. American Journal of Public Health [Internet]. 2014;104:e43–50. Available from: http://ajph.aphapublications.org/doi/10.2105/AJPH.2014.302034

11. Palacios C, Gonzalez L. Is vitamin D deficiency a major global public health problem? The Journal of Steroid Biochemistry and Molecular Biology [Internet]. 2014;144:138–45. Available from: https://linkinghub.elsevier.com/retrieve/pii/S0960076013002331

For peer review only

12. Bischoff-ferrari HA, Giovannucci E, Willet W, Dietrich T, Dawson-hughes B. Estimation of optimal serum concentrations of 25- hydroxyvitamin D for multiple health outcomes. Am J Clin Nutr. 2006;84:18–28.

13. Schwalfenberg GK. No Title a review of the critical role of vitamin D in the function in gof the immune system and the clinical implications of vitamin D deficiency. Mol Nutr Food Res. 2011;55:96–100.

14. Ansary A, Palacios C, De-Regil LM, Peña-Rosas JP. Vitamin D supplementation for women during pregnancy. In: De-Regil LM, editor. Cochrane Data base of Systematic Reviews [Internet]. Chichester, UK: John Wiley& Sons, Ltd; 2010. Available from: http://doi.wiley.com/10.1002/14651858.CD008873

15. Agarwal S, Kovilam O, Agrawal DK. Vitamin D and its impact on maternal-fetal outcomes in pregnancy: A critical review. Critical Reviews in Food Science and Nutrition [Internet]. 2018;58:755–69. Available from: https://www.tandfonline.com/doi/full/10.1080/10408398.2016.1220915

16. Fogacci S, Fogacci F, Banach M, Michos ED, Hernandez A v., Lip GYH, et al. Vitamin D supplementation and incident preeclampsia: A systematic review and meta-analysis of randomized clinical trials. Clinical Nutrition [Internet]. 2020;39:1742–52. Available from: https://linkinghub.elsevier.com/retrieve/pii/S0261561419330274

17. Akbari S, Khodadadi B, Ahmadi SAY, Abbaszadeh S, Shahsavar F. Association of vitamin D level and vitamin D deficiency with risk of preeclampsia: A systematic review and updated meta-analysis. Taiwanese Journal of Obstetrics and Gynecology [Internet]. 2018;57:241–7. Available from: https://linkinghub.elsevier.com/retrieve/pii/S1028455918300317

18. Kassai MS, Cafeo FR, Affonso-Kaufman FA, Suano-Souza FI, Sarni ROS. Vitamin D plasma concentrations in pregnant women and the irpreterm newborns. BMC Pregnancy and Childbirth [Internet]. 2018;18:412. Available from: https://bmcpregnancychildbirth.biomedcentral.com/articles/10.1186/s12884-018-2045-1

19. McDonnell SL, Baggerly KA, Baggerly CA, Aliano JL, French CB, Baggerly LL, et al. Maternal 25(OH)D concentrations ≥40 ng/mL associated with 60% lower preterm birth risk among general obstetrical patients at an urban medical center. Rosenfeld CS, editor. PLOS ONE [Internet]. 2017;12:e0180483. Available from: https://dx.plos.org/10.1371/journal.pone.0180483

20. Fleet JC. The role of vitamin D in the endocrinology control ling calcium home ostasis. Molecular and Cellular Endocrinology [Internet]. 2017;453:36–45. Available from: https://linkinghub.elsevier.com/retrieve/pii/S0303720717302216

21. Sweeney HL, Hammers DW. Muscle Contraction. Cold Spring Harbor Perspectives in Biology [Internet]. 2018;10:a023200. Available from: http://cshperspectives.cshlp.org/lookup/doi/10.1101/cshperspect.a023200

22. Bilezikian JP, Brandi ML, Cusano NE, Mannstadt M, Rejnmark L, Rizzoli R, et al. Management of hypoparathyroidism: Presentand future. Journal of Clinical Endocrinology and Metabolism. Endocrine Society; 2016. p. 2313–24.

For peer review only

23. Kumar N, Singh AK. Maternal serum uric acid and calcium as predictors of hypertensive disorder of pregnancy: A case control study. Taiwanese Journal of Obstetrics and Gynecology. Elsevier Ltd; 2019;58:244–50.

24. Abbasalizadeh S, Abam F, Mirghafourvand M, Abbasalizadeh F, Taghavi S, Hajizadeh K. Comparing levels of vitamin D, calcium and phosphorus in normotensive pregnant women and pregnant women with preeclampsia. Journal of Obstetrics and Gynaecology. Taylor and Francis Ltd.; 2020;40:1069–73.

25. Zhu T, Liu TJ, Ge X, Kong J, Zhang LJ, Zhao Q. High prevalence of maternal vitamin D deficiency in preterm births in northeast China, Shenyang. International Journal of Clinical and Experimental Pathology. E-Century Publishing Corporation; 2015;8:1459–65.

26. Zosky GR, Hart PH, Whitehouse AJO, Kusel MM, Ang W, Foong RE, et al. Vitamin D deficiency at 16 to 20 weeks' gestation is associated with impaired lung function and asthma at 6 years of age. Annals of the American Thoracic Society. American Thoracic Society; 2014;11:571–7.

27. Wang J, Yang F, Mao M, Liu D-H, Yang H-M, Yang S-F. High prevalence of vitamin D and calcium deficiency among pregnant women and the irnewborns in Chengdu, China. World JournalofPediatrics [Internet]. 2010;6:265–7. Available from: http://link.springer.com/10.1007/s12519-010-0224-x

28. Downs SH, Black N. The feasibility of creating a checklist for the assessment of the methodological quality both of randomized and non-randomised studies of health care interventions. Journal of Epidemiology& Community Health [Internet]. 1998;52:377–84. Available from: https://jech.bmj.com/lookup/doi/10.1136/jech.52.6.377

29. Muthuri SG, Venkatesan S, Myles PR, Leonardi-Bee J, al Khuwaitir TSA, al Mamun A, et al. Effectiveness of neuraminidase inhibitors in reducing mortality in patients admitted to hospital with influenza A H1N1 pdm 09 virus infection: A meta-analysisof individual participant data. The Lancet Respiratory Medicine. Lancet Publishing Group; 2014;2:395–404.

30. Guyatt GH, Oxman AD, Sultan S, Glasziou P, Akl EA, Alonso-Coello P, et al. GRADE guidelines: 9. Rating up the quality of evidence. Journal of Clinical Epidemiology. 2011;64:1311–6.

31. Rodrigues CL, Ziegelmann PK. Metanálise: Um Guia Prático. Revista HCPA. 2010;30:436–47.

32. Higgins JPT. Measuring inconsistency in meta-analyses. BMJ [Internet]. 2003;327:557–60. Available from: https://www.bmj.com/lookup/doi/10.1136/bmj.327.7414.557

33. Brasil. Diretrizes Metodológicas: Elaboração de revisão sistemática e metanálise de estudos observacionais comparativos sobre fatores de risco e prognóstico. 2014.

34. Brasil. Ministério da Saúde. Pesquisa nacional de demografia e saúde da criança e da mulher (PNDS 2006): Dimensões do processo reprodutivo e da saúde da criança. Title. 2009.

For peer review only

35. Lício JSA, Fávaro TR, Chaves CRM de M. Anemia em crianças e mulheres indígenas no Brasil: Revisão sistemática. Ciência e Saúde Coletiva. Associação Brasileira de Pos - Graduação em Saúde Coletiva; 2016;21:2571–81.

36. Achebe MM, Gafter-Gvili A. How I treat anemia in pregnancy: Iron, cobalamin, and folate. Blood. American Society of Hematology; 2017;129:940–9.

37. Bezerra AGN, Leal VS, Lira PIC de, Oliveira JS, Costa EC, Menezes RCE de, et al. Anemia e fatores associados em mulheres de idade reprodutiva de um município do Nordeste brasileiro. Revista Brasileira de Epidemiologia [Internet]. 2018;21. Available from: http://www.scielo.br/scielo.php?script=sci_arttext&pid=S1415-790X2018000100400&lng=pt&tlng=pt

38. Bastos Maia S, Costa Caminha M, Lins da Silva S, Rolland Souza A, Carvalho dos Santos C, Batista Filho M. The Prevalence of Vitamin A Deficiency and Associated Factors in Pregnant Women Receiving Prenatal Care at a Reference Maternity Hospital in North easter Brazil. Nutrients [Internet]. 2018;10:1271. Available from: http://www.mdpi.com/2072-6643/10/9/1271

39. Miller R, Spiro A, Stanner S. Micronutrient status and intake in the UK – where might webe in 10 years' time? Nutrition Bulletin [Internet]. 2016;41:14–41. Available from: http://doi.wiley.com/10.1111/nbu.12187

40. Harding KL, Aguayo VM, Webb P. Hidden hunger in South Asia: a review of recent trend sand persistent challenges. Public Health Nutrition [Internet]. 2018;21:785–95. Available from: https://www.cambridge.org/core/product/identifier/S1368980017003202/type/journal_article

41. Kiely ME, Wagner CL, Roth DE. Vitamin D in pregnancy: Where we are and where we should go. J Steroid Biochem Mol Biol. 2020;201:105669.

For peer review only

Table 2. Data extraction worksheet.

Author/ year	Study type	Study location	Age group	Sample type/sample size	Place of residence (rural/urban)	Continent/ World region	Pregnant women (yes/no)	Lactating women (yes/no)	Micronutrients analyzed		Technique used	Cutoff points	Prevalence		Notes
									Calcium	Vit. D			Calcium	Vit. D	

For peer review only

For peer review only - http://bmjopen.bmj.com/site/about/guidelines.xhtml

Figure 1. PRISMA flow diagram.

For peer review only

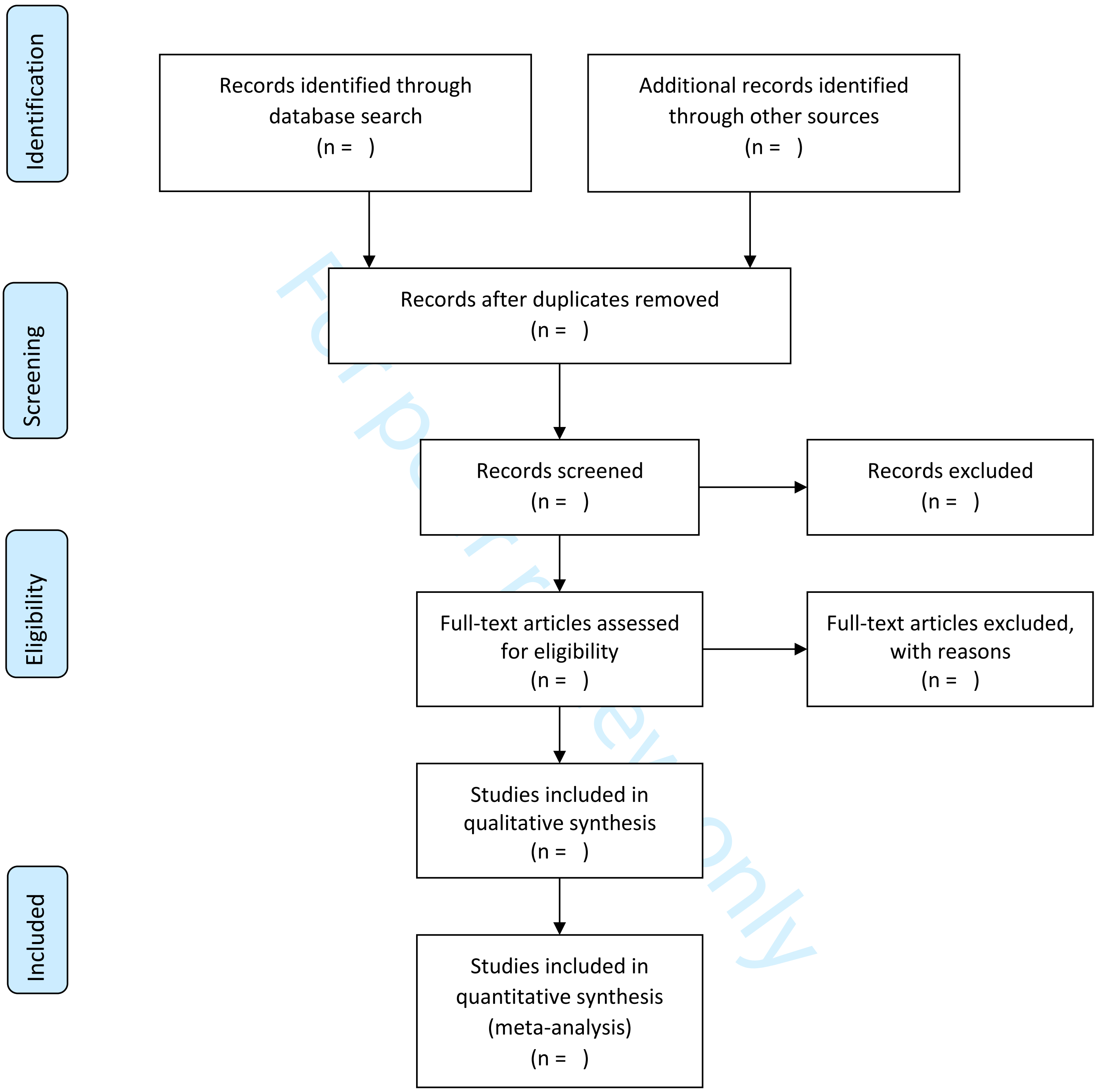
Identification
Records identified through database search (n =)
Additional records identified through other sources (n =)
Screening
Records after duplicates removed (n =)
Records screened (n =)
Records excluded (n =)
Eligibility
Full-text articles assessed for eligibility (n =)
Full-text articles excluded, with reasons (n =)
Included
Studies included in qualitative synthesis (n =)
Studies included in quantitative synthesis (meta-analysis) (n =)

BMJ Open

Prevalence of vitamin D and calcium deficiencies and their health impacts on women of childbearing age: a protocol for systematic review and meta-analysis

Journal:	*BMJ Open*
Manuscript ID	bmjopen-2021-049731.R1
Article Type:	Protocol
Date Submitted by the Author:	13-Oct-2021
Complete List of Authors:	Silveira, Erika; Federal University of Goias, Department of Clinical Medicine; University College London, Department of Clinical Medicine<br>Nogueira, Letícia; Federal University of Goias, Department of Clinical Medicine<br>Castro, Maria ; Federal University of Goias, Department of Clinical Medicine<br>Kac, Gilberto; Federal University of Rio de Janeiro<br>Noll, Priscilla; Goiano Federal Institute, Department of Obstetrics and Gynecology; University of São Paulo, Department of Obstetrics and Gynecology<br>de Oliveira, Cesar; University College London, Epidemiology and Public Health<br>Noll, Matias; Instituto Federal Goiano, Public Health; University of Southern Denmark,
**Primary Subject Heading**:	Nutrition and metabolism
Secondary Subject Heading:	Obstetrics and gynaecology
Keywords:	NUTRITION & DIETETICS, EPIDEMIOLOGY, Protocols & guidelines < HEALTH SERVICES ADMINISTRATION & MANAGEMENT

SCHOLARONE™
Manuscripts

Prevalence of vitamin D and calcium deficiencies and their health impacts on women of childbearing age: a protocol for systematic review and meta-analysis

Erika Aparecida Silveira[1,2]

Letícia de Almeida Nogueira e Moura[1]

Maria Clara Rezende Castro[1]

Gilberto Kac[3]

Priscilla Noll[4,5]

Cesar de Oliveira[2]

Matias Noll[4,6]

[1]Department of Clinical Medicine, Faculty of Medicine, Health Science Post-graduate Program, Federal University of Goias, Goiás, Brazil

[2]Department of Epidemiology & Public Health, Institute of Epidemiology & Health Care, University College London, London, UK

[3]Federal University of Rio de Janeiro, Rio de Janeiro, Brazil

[4]Goiano Federal Institute, Goiás, Brazil

[5]Department of Obstetrics and Gynecology, Medical School, University of São Paulo, São Paulo, Brazil

[6]Department of Sports Science and Clinical Biomechanics, University of Southern Denmark, Odense, Denmark

Correspondent author: Erika A. Silveira: erikasil@terra.com.br

For peer review only

ABSTRACT

Introduction: No systematic reviews has synthesized data on the available evidence to determine the prevalence of calcium and vitamin D deficiencies as a public health problem globally. Therefore, this study presents a protocol for conducting a review and meta-analysis to estimate the prevalence of calcium and vitamin D serum deficiencies in women of childbearing age and stratify these data by age group, urban and rural area, world region, and pregnant/non-pregnant women whenever possible. **Methods and analysis:** The systematic review protocol involves conducting a literature search in the following databases: PubMed, LILACS, Embase, Scopus, and Web of Science. The selected articles will be checked thoroughly, including the references to include gray literature. Cross-sectional studies and baseline data from cohort studies or clinical and community trials conducted with women of childbearing age with representative probabilistic sampling will be included. Two independent researchers will be responsible for article selection and data extraction, and discrepancies, if any, will be dealt with by a third reviewer. Methodological quality and risk of bias will be analyzed using the Grading of Recommendations, Assessment, Development, and Evaluations, and JBI check-list, respectively. The heterogeneity of the estimates between studies will also be evaluated. The results will be presented in a report format that will systematically organize evidence on the prevalence of the most critical micronutrient deficiencies in women of childbearing age, emphasizing calcium and vitamin D levels in pregnant and non-pregnant women. Dissemination of the key findings from the systematic review will help identify priorities for action, establish dietary guidelines, develop health-related public policies, and reduce and combat micronutrient deficiencies among women of childbearing age and their children.

Ethics and dissemination: The protocol was written according to the Preferred Reporting Items for Systematic Reviews and Meta-Analyses. No ethical approval is required.

Systematic review registration: PROSPERO CRD42020207850.

Keywords: Micronutrients, Calcium, Vitamin D, Childbearing age, Systematic Review Protocol, Nutritional Deficiencies, Epidemiology

For peer review only

Strengths and limitations of this study

► This systematic review intends to estimate the prevalence of calcium and vitamin D serum deficiencies in women of childbearing age and stratify these data by age group, urban and rural area, world region, and pregnant/non-pregnant women whenever possible;

► The following five databases will be included to identify scientific articles: PubMed, LILACS, Embase, Scopus, and Web of Science;

► Meta-analysis will be evaluated using fixed-effects or random-effects methods according to the studies' homogeneity whenever possible;

► Potential heterogeneity of measurements and assessments of micronutrient deficiency in research may be a limitation.

For peer review only

INTRODUCTION

Nutritional deficiencies have multiple etiologies related to factors such as nutrient loss, malabsorption, inadequate intake, and even ignorance of the importance of nutritional care during pregnancy[1]. Women of childbearing age are part of the highly vulnerable population and often experience these deficiencies, especially during pregnancy [2–5]. Besides, maternal nutritional status influences the pregnancy outcomes and child's health and, as it is necessary to supply the needs of both the mother and the fetus[3–5].

Vitamin D deficiency is considered a significant public health problem and affects approximately one billion people worldwide [6,7]. However, there are significant data gaps in measuring this problem magnitude in many countries[8]. The estimated prevalence of vitamin D deficiency (20ng/ml) was around 37% in Canada and 40% in Europe[9]. A systematic review with a meta-analysis of observational studies found a significant association between low vitamin D levels and all-cause mortality [10]. The causal relationships of vitamin D with diseases are less clear, and they should only refer to meta-analyses of randomized trials [10–14]. Specifically for pregnant women, systematic reviews with meta-analysis of randomized clinical trials found that vitamin D supplementation can effectively prevent pre-eclampsia [11] and reduce maternal insulin resistance [12]. Furthermore, it is associated with high circulating levels of 25(OH)D, birth weight and length [13], and fetal or neonatal mortality [14]

Vitamin D levels interfere with calcium metabolism[15]. Calcium is a micronutrient that plays an essential role in muscle contraction and regulates water balance in cells[16]. Hypocalcemia symptoms, characterized by low calcium levels, are variable, ranging from mild disorders such as perioral paresthesia to severe life-threatening ones such as cardiac arrhythmias[17]. In pregnant women, this imbalance is characterized by a lower serum concentration and a higher concentration in the cells, thereby causing increased blood pressure in women with pre-eclampsia[18,19].

Previous studies have evaluated the prevalence of vitamin D and calcium deficiencies in women of childbearing age[20–22]. However, at present, there is no systematic review that synthesizes the state of art of this relevant public health problem nationally and globally. Therefore, this study aimed to estimate the prevalence of calcium and vitamin D serum deficiencies in women of childbearing age. In addition, the data will

For peer review only

be stratified by age group, urban and rural areas, geographical location, and pregnant/non-pregnant women through a systematic review followed by a meta-analysis.

METHODS AND ANALYSIS

This systematic review and meta-analysis will be written according to the Preferred Reporting Items for Systematic Reviews and Meta-Analyses Protocols (PRISMA). The protocol for this systematic review was registered on the Prospero platform (CRD42020207850). This review follows the Population, Intervention, Comparison, and Outcome (PICO) structure, with the population (P) women of childbearing age, the intervention (I) no intervention on this study, the comparison (C) being conducted between subgroups in childbearing age as pregnant and not pregnant, and the outcome (O) being calcium and vitamin D deficiencies. Therefore, our systematic review will be conducted based on the following research question: 'What is the prevalence of calcium and vitamin D serum deficiencies in women of childbearing age?'

Search strategy and databases

In December 2021, a systematic review of the literature will be conducted to identify and collect bibliographic data in the following databases: PubMed (National Library of Medicine, United States), LILACS (Latin American and Caribbean Health Sciences Literature), Embase, Scopus, and Web of Science databases (**Figure 1**). The search strategy will be complemented by screening the reference list of the included studies and relevant systematic reviews.

Different search strategies will include Medical Subject Heading (MeSH) terms and relevant keywords related to vitamin D deficiency and specific terms related to calcium deficiency to ensure that all articles of interest are identified using all the descriptors and Boolean operators of this systematic review protocol (**Supplementary Table**).

Eligibility criteria

Inclusion criteria

For peer review only

- Studies that provides data on the prevalence of calcium and/or vitamin D serum deficiency in women of childbearing age (15-49 years old or menarche and menopause).
- Studies with representative population-based samples in hospitals, health centers, or outpatient clinics.
- Prevalence data on women of different age groups, such as adolescents, pregnant women, lactating women, and premenopausal adult women.
- Studies with cross-sectional design and also data's from longitudinal studies (cohort studies) or intervention studies such as clinical trials or community trials since they have prevalence information in a specific point of time. included analysis data from some point in the cohort study time that characterizes prevalence.
- Articles in English, Portuguese, and Spanish language were selected.

Exclusion criteria

- Opinion articles, comments, or editorials.
- Duplicates: The most comprehensive one that reports the largest sample size will be considered for studies published as more than one article.
- Studies with primary data not accessible even after request.
- Case-control studies, narrative reviews, and case series.
- Studies conducted among athlete women of any sports modality.
- Studies conducted among women with specific diseases:
  - *Autoimmune diseases:* lupus, psoriasis, thyroiditis, rheumatoid arthritis, multiple sclerosis
  - *Eating disorders:* Anorexia, Bulimia
  - *Hematological diseases:* Thalassemia, Sickle Cell Disease
  - *Respiratory diseases:* COPD, Asthma, Pneumonia, Respiratory Infections, Tuberculosis
  - *Chronic diseases such as:* heart failure, kidney failure, liver diseases, chronic kidney disease, cardiac diseases, nephrotic syndrome, AIDS, inflammatory bowel disease, hypothyroidism, sepsis, cancer
  - *Genetic diseases and syndromes:* Mutation in the vitamin D receptor, Cystic Fibrosis, Prader-Willi

For peer review only

- *Neurological or psychiatric illnesses:* Epilepsy or use of antiepileptic medication, ADHD, schizophrenia
- *Trauma, post-surgical:* Burns, Post-bariatric, in the treatment of recent fractures or orthopedic/osteoarticular diseases
- Patients in intensive care, urgency and emergency and palliative care

- Small number of participants (< 50)
- Studies conducted among indigenous women.

Reviewers' training

The authors responsible for assessing article eligibility will be trained on using the inclusion and exclusion criteria. Thus, an eligibility test will be conducted on 50 titles and abstracts before codifying the articles. They will also receive training on instruments to assess the risk of bias used on five articles that have not been included. Rayyan and Mendeley software will be used for the selection steps.

Review process

After completing the search strategy, the identified articles will be gathered and imported into Mendeley software. Duplicate articles will be deleted. The articles will be selected by two independent reviewers (LM and MC). The titles will be read in the first stage, and the abstracts in the second stage. Finally, in the third stage, the entire article will be read. Disagreements between reviewers will be assessed by a third reviewer (EAS). Eligibility will be determined according to the inclusion and exclusion criteria. The percentage of agreement and the Kappa coefficient will be calculated to assess reliability between reviewers' assessments. At the end of the process, the selected articles will be included in the systematic review.

Data extraction and risk of bias assessment

Data extraction will be performed using a spreadsheet that considers the following aspects: author/year, type of study, place of study, age group, type of sample/sample size, place of residence (urban/rural), continent/world region (Asia, Europe, Africa, Latin America, North America, and Oceania), a pregnant woman (yes/no), lactating (yes/no), micronutrients analyzed, the technique used, cutoff points, and results, i.e., prevalence/impact of calcium or vitamin D deficiencies (**Table 1**).

For peer review only

In this study, we will use the Joanna Briggs Institute development tool of a critical appraisal tool for use in systematic reviews addressing questions of prevalence[23]. This tool has excellent methodological rigor, handling essential items related to the methodological quality of the studies[24]. To ensure the quality of the study, the tool consists of ten questions that assess criteria such as representativeness and sample size, recruitment of participants, and data analysis[23].

The Grading of Recommendations, Assessment, Development, and Evaluations (GRADE) will be used to assess the strength of evidence[25]. The quality of evidence will be classified into four grades: high, moderate, low, or very low[25].

The data will be extracted and evaluated by two independent reviewers (LM and MC). Disagreements will be resolved by a third reviewer (MN). To obtain relevant data not described in the manuscript, a researcher (MC) will contact the article's authors. The authors of the included studies' potential conflicts of interest and ethical information will also be described.

Patient and Public Involvement

No patient involved

Statistical analysis

Meta-analysis will be performed using fixed-effects or random-effects methods, depending on the heterogeneity between studies. Chi-square test with a significance level ($p < 0.05$) will be used to assess heterogeneity, and the I-square statistic (I^2) will be used to determine the magnitude of inconsistency. I^2 results above 75% will indicate a high heterogeneity, those between 25% and 75% will characterize moderate heterogeneity, and those below 25% will indicate low heterogeneity[26–28].

According to the available literature, sensitivity and estimation of the prevalence of vitamin D and calcium deficiencies will be analyzed. The data will be stratified according to region, age, and urban/rural area subgroups. If applicable, meta-regression will be performed to explain the studies' heterogeneity considering $p < 0.05$ for each stratification subgroup. We will estimate the pooled prevalence's by obtaining the following information from each study: the number of women with calcium and vitamin D deficiencies and sample size total. Analyses will be conducted using R through the

For peer review only - http://bmjopen.bmj.com/site/about/guidelines.xhtml

package Meta. The results will be presented as prevalence with 95% confidence interval (CI95%).

The primary outcome of the present study is the prevalence of serum calcium and vitamin D deficiencies in women of childbearing age.

DISCUSSION

Nutritional deficiencies affect pregnant and non-pregnant women, especially those living in poorer regions like the Brazilian northeast[29–33]. Thus, such nutritional deficiencies need to be reviewed nationally and globally as public health problems [34,35]. Therefore, it is essential to estimate the extension of this problem. To the best of our knowledge, there are no systematic reviews and meta-analyses that synthesize data on the available evidence to assess the extent of the prevalence of calcium and vitamin D deficiencies in women of childbearing age.

Obstacles in implementing policies and public health programs to combat nutritional deficiencies can be exacerbated by inequalities in health service provision and access [36]. Therefore, it is essential to gather, organize, and systematize the available data on the prevalence of the main vitamin and mineral deficiencies to develop intervention plans, list action priorities, and improve existing public health policies for women of childbearing age.

This systematic review protocol is important to improve knowledge on global and Brazilian realities on the prevalence of vitamin D and calcium deficiency in this population group. This study is expected to contribute to suggestions for priority action strategies and the development of public health policies to minimize the problem and prevent the occurrence of new cases and assist in establishing intervention protocols to reduce the main deficiencies according to the priorities indicated in the final report. However, some limitations of this research need to be highlighted. First, the potential heterogeneity of measurements and assessments of micronutrient deficiency in research. Second, studies may have used different methods, making it difficult to conduct a meta-analysis.

Women of childbearing age, especially pregnant women, are part of a highly vulnerable group that develops nutritional deficiencies, and the maternal nutritional status influences gestational outcomes and the child's health. Considering the above, assessing

For peer review only

the prevalence of vitamin D and calcium deficiencies could help identify knowledge gaps on the magnitude of these deficiencies, warranting further studies. From this perspective, disseminating the results of this systematic review protocol will be helpful and vital to encourage research and identifying priorities, establishing dietary guidelines, and reducing and combating micronutrient deficiencies in this population.

Ethics statements

Patient consent for publication

Not required. No patient was involved.

Ethics and Dissemination

A systematic review does not require ethical approval since it synthesizes publicly available literature and will not include individual patient data. The results of the scoping review will be submitted for publication to a scientific journal and presented at relevant conferences.

Acknowledgment

The authors thank CNPq and IF Goiano for providing partial support.

Funding sources/sponsors

This study received support from the This study received support from the MS-SCTIE-Decit/*CNPq* Nº 442932/2019-7. Dr Cesar de Oliveira is supported by the Economic and Social Research Council (Grant ES/T008822/1)

Conflicts of interest

The authors of this systematic review and meta-analysis declare no conflicts of interest. This review will analyze whether the studies report a possible conflict of interest and approval by the Research Ethics Committee (ELIA et al., 2016). It will also verify if the studies followed the ethical requirements recommended in the Declaration of Helsinki, respecting all the ethical principles established in resolution 466/2012 of the

For peer review only

National Health Council (BRASIL, 2012) or the norms and ethical resolutions in force at the time of data collection.

Contributorship statement

Conceptualization, ES, LA, MC, and MN; methodology, ES, LA, MC, and MN; formal analysis, ES, LA, MC, and MN; investigation, ES, LA, MC, GK, PN, CO, and MN; resources, ES.; writing—original draft preparation, ES, LA, MC, GK, PN, CO, and MN; writing—review and editing, ES, LA, MC, GK, PN, CO, and MN; visualization ES, LA, MC, GK, PN, CO, and MN; supervision, MN, and ES.; funding acquisition, ES. All authors have read and agreed to the published version of the manuscript.

REFERENCES

1 Miranda VIA, da Silva Dal Pizzol T, Silveira MPT, *et al.* The use of folic acid, iron salts and other vitamins by pregnant women in the 2015 Pelotas birth cohort: is there socioeconomic inequality? *BMC Public Health* 2019;**19**:889. doi:10.1186/s12889-019-7269-0

2 Barger MK. Maternal Nutrition and Perinatal Outcomes. *Journal of Midwifery & Women's Health* 2010;**55**:502–11. doi:10.1016/j.jmwh.2010.02.017

3 Iqbal S, Ekmekcioglu C. Maternal and neonatal outcomes related to iron supplementation or iron status: a summary of meta-analyses. *The Journal of Maternal-Fetal & Neonatal Medicine* 2019;**32**:1528–40. doi:10.1080/14767058.2017.1406915

4 Obeid R, Murphy M, Solé-Navais P, *et al.* Cobalamin Status from Pregnancy to Early Childhood: Lessons from Global Experience. *Advances in Nutrition: An International Review Journal* 2017;**8**:971–9. doi:10.3945/an.117.015628

5 Nauman, Nuzhat ; Jalali, Samina; Shami, Sajjad ; Rafiq, Shireen; Große, Greta; Hilger, Alina C .; Zhang, Rhong; Mansoor, Saira; Ludwig, Michael L.; Reutter H. Low maternal folate concentrations and maternal MTHFR C677T polymorphism are associated with an increased risk for neural tube defects in offspring: a case-control study among Pakistani case and control mothers. *Asia Pac J Clin Nutr* 2018;**27**:253–60. doi:10.6133/apjcn.032017.10

6 Mithal A, Wahl DA, Bonjour J-P, *et al.* Global vitamin D status and determinants of hypovitaminosis D. *Osteoporosis International* 2009;**20**:1807–20. doi:10.1007/s00198-009-0954-6

7 Martins MEP, Esmeraldo CUP, Sabiá JPD, *et al.* Vitamin D Postpartum Concentrations: Relationship with Nutritional Condition and Morbidities during Pregnancy. *Journal of Pregnancy* 2018;**2018**:1–6. doi:10.1155/2018/1070528

For peer review only

8 Roth DE, Abrams SA, Aloia J, *et al.* Global prevalence and disease burden of vitamin D deficiency: a roadmap for action in low- and middle-income countries. *Annals of the New York Academy of Sciences* 2018;**1430**:44–79. doi:10.1111/nyas.13968

9 Amrein K, Scherkl M, Hoffmann M, *et al.* Vitamin D deficiency 2.0: an update on the current status worldwide. *European Journal of Clinical Nutrition* 2020;**74**:1498–513. doi:10.1038/s41430-020-0558-y

10 Garland CF, Kim JJ, Mohr SB, *et al.* Meta-analysis of All-Cause Mortality According to Serum 25-Hydroxyvitamin D. *American Journal of Public Health* 2014;**104**:e43–50. doi:10.2105/AJPH.2014.302034

11 Fogacci S, Fogacci F, Banach M, *et al.* Vitamin D supplementation and incident preeclampsia: A systematic review and meta-analysis of randomized clinical trials. *Clinical Nutrition* 2020;**39**:1742–52. doi:10.1016/j.clnu.2019.08.015

12 Gallo S, McDermid JM, Al-Nimr RI, *et al.* Vitamin D Supplementation during Pregnancy: An Evidence Analysis Center Systematic Review and Meta-Analysis. *Journal of the Academy of Nutrition and Dietetics* 2020;**120**:898-924.e4. doi:10.1016/j.jand.2019.07.002

13 Pérez-López FR, Pasupuleti V, Mezones-Holguin E, *et al.* Effect of vitamin D supplementation during pregnancy on maternal and neonatal outcomes: a systematic review and meta-analysis of randomized controlled trials. *Fertility and Sterility* 2015;**103**:1278-1288.e4. doi:10.1016/j.fertnstert.2015.02.019

14 Bi WG, Nuyt AM, Weiler H, *et al.* Association Between Vitamin D Supplementation During Pregnancy and Offspring Growth, Morbidity, and Mortality. *JAMA Pediatrics* 2018;**172**:635. doi:10.1001/jamapediatrics.2018.0302

15 Fleet JC. The role of vitamin D in the endocrinology controlling calcium homeostasis. *Molecular and Cellular Endocrinology* 2017;**453**:36–45. doi:10.1016/j.mce.2017.04.008

16 Sweeney HL, Hammers DW. Muscle Contraction. *Cold Spring Harbor Perspectives in Biology* 2018;**10**:a023200. doi:10.1101/cshperspect.a023200

17 Bilezikian JP, Brandi ML, Cusano NE, *et al.* Management of Hypoparathyroidism: Present and Future. *The Journal of Clinical Endocrinology & Metabolism* 2016;**101**:2313–24. doi:10.1210/jc.2015-3910

18 Kumar N, Singh AK. Maternal serum uric acid and calcium as predictors of hypertensive disorder of pregnancy: A case control study. *Taiwanese Journal of Obstetrics and Gynecology* 2019;**58**:244–50. doi:10.1016/j.tjog.2019.01.014

19 Abbasalizadeh S, Abam F, Mirghafourvand M, *et al.* Comparing levels of vitamin D, calcium and phosphorus in normotensive pregnant women and pregnant women with preeclampsia. *Journal of Obstetrics and Gynaecology* 2020;**40**:1069–73. doi:10.1080/01443615.2019.1678575

20 Zhu T, Liu T-J, Ge X, *et al.* High prevalence of maternal vitamin D deficiency in preterm births in northeast China, Shenyang. *International journal of clinical and experimental pathology* 2015;**8**:1459–65.http://www.ncbi.nlm.nih.gov/pubmed/4396334

21 Zosky GR, Hart PH, Whitehouse AJO, *et al.* Vitamin D Deficiency at 16 to 20 Weeks' Gestation Is Associated with Impaired Lung Function and Asthma at 6 Years of Age.

For peer review only

Annals of the American Thoracic Society 2014;**11**:571–7. doi:10.1513/AnnalsATS.201312-423OC

22 Wang J, Yang F, Mao M, *et al.* High prevalence of vitamin D and calcium deficiency among pregnant women and their newborns in Chengdu, China. *World Journal of Pediatrics* 2010;**6**:265–7. doi:10.1007/s12519-010-0224-x

23 Munn Z, Moola S, Riitano D, *et al.* The development of a critical appraisal tool for use in systematic reviews addressing questions of prevalence. *International Journal of Health Policy and Management* 2014;**3**:123–8. doi:10.15171/ijhpm.2014.71

24 Migliavaca CB, Stein C, Colpani V, *et al.* Quality assessment of prevalence studies: a systematic review. *Journal of Clinical Epidemiology* 2020;**127**:59–68. doi:10.1016/j.jclinepi.2020.06.039

25 Guyatt GH, Oxman AD, Sultan S, *et al.* GRADE guidelines: 9. Rating up the quality of evidence. *Journal of Clinical Epidemiology* 2011;**64**:1311–6. doi:10.1016/j.jclinepi.2011.06.004

26 Rodrigues, Caroline Legramanti; Ziegelman PKlarmann. META-ANALYSIS: A PRACTICAL GUIDE. *Rev HCPA* 2010;**30**:436–47.

27 Higgins JPT. Measuring inconsistency in meta-analyses. *BMJ* 2003;**327**:557–60. doi:10.1136/bmj.327.7414.557

28 Brasil. Ministério da Saúde. Diretrizes metodológicas : elaboração de revisão sistemática e metanálise de ensaios clínicos randomizados. 2012.

29 Saúde. BrasilM da. Pesquisa nacional de demografia e saúde da criança e da mulher (PNDS 2006): Dimensões do processo reprodutivo e da saúde da criança. 2009.

30 Lício JSA, Fávaro TR, Chaves CRM de M. Anemia em crianças e mulheres indígenas no Brasil: revisão sistemática. *Ciência & Saúde Coletiva* 2016;**21**:2571–81. doi:10.1590/1413-81232015218.00532015

31 Achebe MM, Gafter-Gvili A. How I treat anemia in pregnancy: iron, cobalamin, and folate. *Blood* 2017;**129**:940–9. doi:10.1182/blood-2016-08-672246

32 Bezerra AGN, Leal VS, Lira PIC de, *et al.* Anemia e fatores associados em mulheres de idade reprodutiva de um município do Nordeste brasileiro. *Revista Brasileira de Epidemiologia* 2018;**21**. doi:10.1590/1980-549720180001

33 Bastos Maia S, Costa Caminha M, Lins da Silva S, *et al.* The Prevalence of Vitamin A Deficiency and Associated Factors in Pregnant Women Receiving Prenatal Care at a Reference Maternity Hospital in Northeastern Brazil. *Nutrients* 2018;**10**:1271. doi:10.3390/nu10091271

34 Miller R, Spiro A, Stanner S. Micronutrient status and intake in the UK - where might we be in 10 years' time? *Nutrition Bulletin* 2016;**41**:14–41. doi:10.1111/nbu.12187

35 Harding KL, Aguayo VM, Webb P. Hidden hunger in South Asia: a review of recent trends and persistent challenges. *Public Health Nutrition* 2018;**21**:785–95. doi:10.1017/S1368980017003202

36 Kiely ME, Wagner CL, Roth DE. Vitamin D in pregnancy: Where we are and where we should go. *The Journal of Steroid Biochemistry and Molecular Biology* 2020;**201**:105669. doi:10.1016/j.jsbmb.2020.105669

For peer review only

For peer review only

Table 1. Data extraction worksheet.

Author/ year	Study type	Study location	Age group	Sample type/sample size	Place of residence (rural/urban)	Continent/ World region	Pregnant women (yes/no)	Lactating women (yes/no)	Micronutrients analyzed		Technique used	Cutoff points	Prevalence		Notes
									Calcium	Vit. D			Calcium	Vit. D	

For peer review only

Figure 1. PRISMA flow diagram.

For peer review only

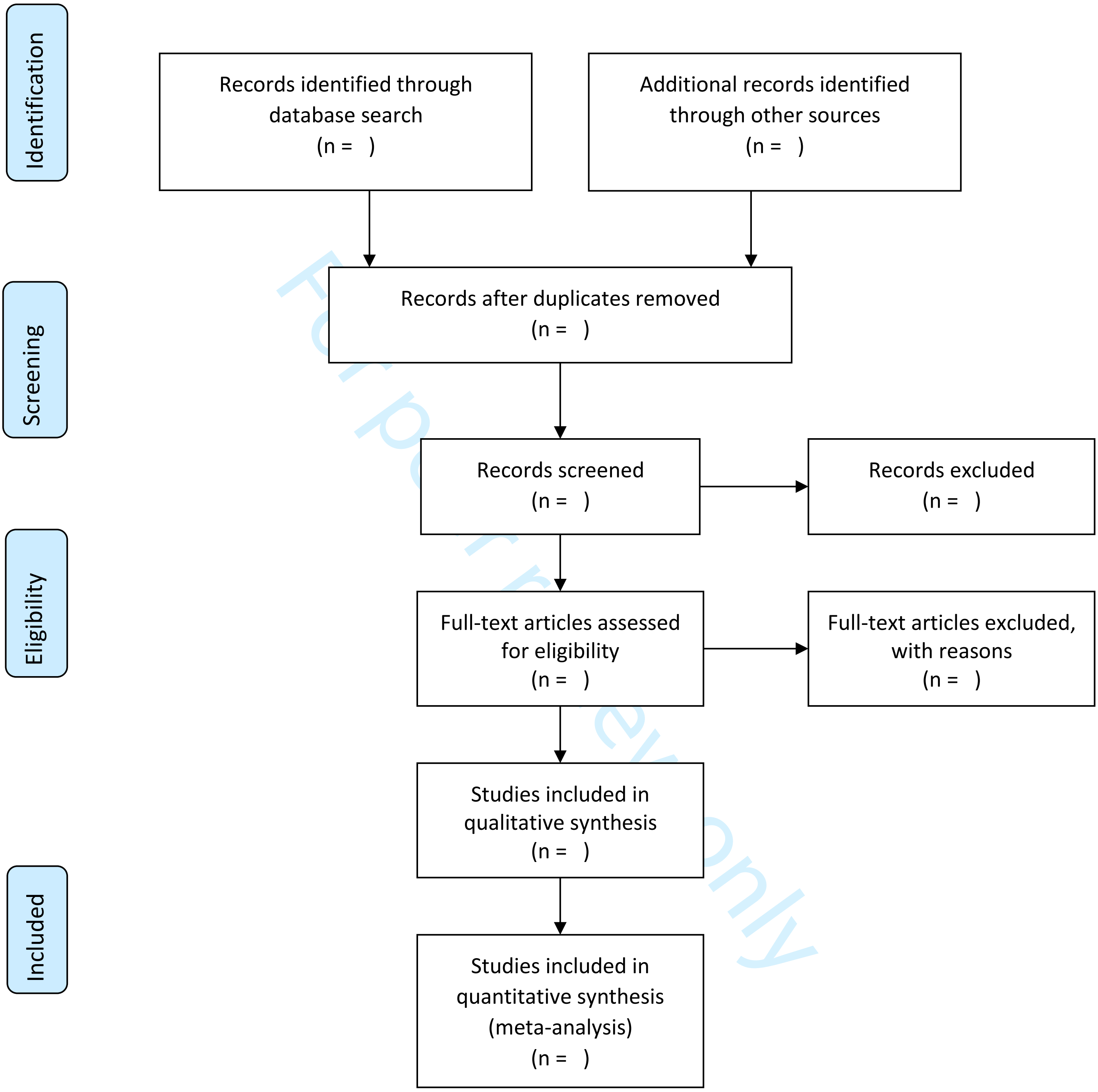
Identification
Screening
Eligibility
Included
Records identified through database search (n =)
Additional records identified through other sources (n =)
Records after duplicates removed (n =)
Records screened (n =)
Records excluded (n =)
Full-text articles assessed for eligibility (n =)
Full-text articles excluded, with reasons (n =)
Studies included in qualitative synthesis (n =)
Studies included in quantitative synthesis (meta-analysis) (n =)

Supplementary Table. Search terms selected according to specific nomenclature for each database

**MEDLINE**	(("Micronutrients" [Title/Abstract] OR "Micronutrients" [MeSH Terms] OR "Deficiency Diseases" [Title/Abstract] OR "Deficiency Diseases" [MeSH Terms] OR "Avitaminosis" [Title/Abstract] OR "Avitaminosis" [MeSH Terms] OR "Deficiencies vitamin" [Title/Abstract] OR "Deficiencies vitamin" [MeSH Terms] OR "Deficiency vitamin" [Title/Abstract] OR "Vitamin deficiency" [Title/Abstract] OR "Vitamin Deficiencies" [Title/Abstract] OR "Serum Levels" [Title/Abstract] OR "Nutritional Status" [Title/Abstract] OR "Nutritional Status" [MeSH Terms] OR "Reduced Level" [Title/Abstract] OR "Reduced Level" [MeSH Terms]) AND ("Women" [Title/Abstract] OR "Women" [MeSH Terms] OR "Girl" [Title/Abstract] OR "Girls" [Title/Abstract] OR "Woman" [Title/Abstract] OR "Adolescent" [Title/Abstract] OR "Adolescent" [MeSH Terms] OR "Teen" [Title/Abstract] OR "Teens" [Title/Abstract] OR "Teenager" [Title/Abstract] OR "Pregnancy" [Title/Abstract] OR "Pregnancy" [MeSH Terms] OR "Gestation" [Title/Abstract] OR "Pregnancies" [Title/Abstract])) AND ("Vitamin D" [Title/Abstract] OR "Vitamin D" [MeSH Term] OR "Vitamin D Deficiency" [Title/Abstract] OR "Vitamin D Deficiency" [MeSH Term] OR "deficiencies vitamin d" [Title/Abstract] OR "Vitamin D Deficiencies" [Title/Abstract] OR "Vitamin D Deficiencies" [MeSH Term] OR "Calcium" [Title/Abstract] OR "Calcium" [MeSH Term] OR "Hypocalcemia" [Title/Abstract] OR "Hypocalcemia" [MeSH Term] OR "Hypocalcemias" [Title/Abstract])
**Embase**	('micronutrients':ab,ti OR 'deficiency diseases':ab,ti OR 'avitaminosis':ab,ti OR 'avitaminoses':ab,ti OR 'deficiencies vitamin':ab,ti OR 'deficiency vitamin':ab,ti OR 'vitamin deficiency':ab,ti OR 'vitamin deficiencies':ab,ti OR 'serum levels':ab,ti OR 'nutritional status':ab,ti OR 'reduced level':ab,ti) AND ('women':ab,ti OR 'girl':ab,ti OR 'girls':ab,ti OR 'woman':ab,ti OR 'adolescent':ab,ti OR 'teen':ab,ti OR 'teens':ab,ti OR 'teenager':ab,ti OR 'pregnancy':ab,ti OR 'gestation':ab,ti OR 'pregnancies':ab,ti) AND ('vitamin d':ab,ti OR 'vitamin d deficiency':ab,ti OR 'deficiencies vitamin d':ab,ti OR 'vitamin d deficiencies':ab,ti OR 'calcium':ab,ti OR 'hypocalcemia':ab,ti OR 'hypocalcemias':ab,ti)
**Scopus**	(TITLE-ABS ("Micronutrients" OR "Deficiency Diseases" OR "Avitaminosis" OR "Avitaminoses" OR "Deficiencies, Vitamin" OR "Deficiency Vitamin" OR "Vitamin Deficiency" OR "Vitamin Deficiencies" OR "Serum Levels" OR "Nutritional Status" OR "Reduced Level")) AND (TITLE-ABS ("Women" OR "Girl" OR "Girls" OR "Woman" OR "Adolescent" OR "Teen" OR "Teens" OR "Teenager" OR "Pregnancy" OR "Gestation" OR "Pregnancies")) AND (TITLE-ABS ("Vitamin D" OR "Vitamin D Deficiency" OR "Deficiencies, Vitamin D" OR "Vitamin D Deficiencies" OR "Calcium" OR "Hypocalcemia" OR "Hypocalcemias"))
**Web of Science**	(TI= "Micronutrients" OR AB= "Micronutrients" OR TI="Deficiency Diseases" OR AB= "Deficiency Diseases" OR TI= "Avitaminosis" OR AB= "Avitaminosis" OR TI= "Avitaminoses" OR AB= "Avitaminoses" OR TI= "deficiencies vitamin" OR AB= "deficiencies vitamin" OR TI= "Deficiency Vitamin" OR AB= "Deficiency Vitamin" OR TI= "Vitamin Deficiency" OR AB="Vitamin Deficiency" OR TI= "Vitamin Deficiencies" OR AB="Vitamin Deficiencies" OR TI= "Serum Levels" OR AB= "Serum Levels" OR TI="Nutritional Status" OR AB="Nutritional Status" OR TI="Reduced Level" OR AB="Reduced Level") AND (TI="Women" OR AB= "Women" OR TI="Girl" OR AB="Girl" OR TI="Girls" OR AB="Girls"

	OR TI="Woman" OR AB="Woman" OR TI="Adolescent" OR AB= "Adolescent" OR TI="Teen" OR AB="Teen" OR TI="Teens" OR AB="Teens" OR TI="Teenager" OR AB="Teenager" OR TI="Pregnancy" OR AB="Pregnancy" OR TI="Gestation" OR AB="Gestation" OR TI="Pregnancies" OR AB="Pregnancies") AND (TI="Vitamin D" OR AB="Vitamin D" OR TI="Vitamin D Deficiency" OR AB= "Vitamin D Deficiency" OR TI="deficiencies vitamin d" OR AB="deficiencies vitamin d" OR TI="Vitamin D Deficiencies" OR AB="Vitamin D Deficiencies" OR TI="Calcium" OR AB="Calcium" OR TI="Hypocalcemia" OR AB= "Hypocalcemia" OR TI="Hypocalcemias" OR AB= "Hypocalcemias")
**LILACS**	(tw:((tw:("Micronutrients")) OR (tw:("Deficiency Diseases")) OR (tw:("Avitaminosis")) OR (tw:("Avitaminoses")) OR (tw:("deficiencies vitamin")) OR (tw:("Deficiency Vitamin")) OR (tw:("Vitamin Deficiency")) OR (tw:("Vitamin Deficiencies")) OR (tw:("Serum Levels")) OR (tw:("Nutritional Status")) OR (tw:("Reduced Level")))) AND (tw:(Women OR Girl OR Girls OR Woman OR Adolescent OR Teen OR Teens OR Teenager OR Pregnancy OR Gestation OR Pregnancies)) AND (tw:(("Vitamin D") OR ("Vitamin D Deficiency") OR ("deficiencies vitamin d") OR ("Vitamin D Deficiencies") OR "Calcium" OR "Hypocalcemia" OR "Hypocalcemias"))

For peer review only

Table
PRISMA-P (preferred reporting items for systematic review and meta-analysis protocols) 2015 checklist: recommended items to address in a systematic review protocol

Section and topic	Item No	Checklist item	Page
**Administrative information**			
Identification	1a	Identify the report as a protocol of a systematic review	1
Update	1b	If the protocol is for an update of a previous systematic review, identify as such	NA
Registration	2	If registered, provide the name of the registry (such as PROSPERO) and registration number	2
Authors:			
Contact	3a	Provide name, institutional affiliation, e-mail address of all protocol authors; provide physical mailing address of corresponding author	1
Contributions	3b	Describe contributions of protocol authors and identify the guarantor of the review	1
Amendments	4	If the protocol represents an amendment of a previously completed or published protocol, identify as such and list changes; otherwise, state plan for documenting important protocol amendments	NA
Support:			
Sources	5a	Indicate sources of financial or other support for the review	11
Sponsor	5b	Provide name for the review funder and/or sponsor	11
Role of sponsor or funder	5c	Describe roles of funder(s), sponsor(s), and/or institution(s), if any, in developing the protocol	11
**Introduction**			

For peer review only - http://bmjopen.bmj.com/site/about/guidelines.xhtml

Section and topic	Item No	Checklist item	Page
Rationale	6	Describe the rationale for the review in the context of what is already known	4
Objectives	7	Provide an explicit statement of the question(s) the review will address with reference to participants, interventions, comparators, and outcomes (PICO)	4/5
**Methods**			
Eligibility criteria	8	Specify the study characteristics (such as PICO, study design, setting, time frame) and report characteristics (such as years considered, language, publication status) to be used as criteria for eligibility for the review	5
Information sources	9	Describe all intended information sources (such as electronic databases, contact with study authors, trial registers or other grey literature sources) with planned dates of coverage	5
Search strategy	10	Present draft of search strategy to be used for at least one electronic database, including planned limits, such that it could be repeated	5/6/7
Study records:			
Data management	11a	Describe the mechanism(s) that will be used to manage records and data throughout the review	8
Selection process	11b	State the process that will be used for selecting studies (such as two independent reviewers) through each phase of the review (that is, screening, eligibility and inclusion in meta-analysis)	8

Section and topic	Item No	Checklist item	Page
Data collection process	11c	Describe planned method of extracting data from reports (such as piloting forms, done independently, in duplicate), any processes for obtaining and confirming data from investigators	8
Data items	12	List and define all variables for which data will be sought (such as PICO items, funding sources), any pre-planned data assumptions and simplifications	8
Outcomes and prioritization	13	List and define all outcomes for which data will be sought, including prioritization of main and additional outcomes, with rationale	8
Risk of bias in individual studies	14	Describe anticipated methods for assessing risk of bias of individual studies, including whether this will be done at the outcome or study level, or both; state how this information will be used in data synthesis	8/9
Data synthesis	15a	Describe criteria under which study data will be quantitatively synthesised	9
	15b	If data are appropriate for quantitative synthesis, describe planned summary measures, methods of handling data and methods of combining data from studies, including any planned exploration of consistency (such as I^2, Kendall's τ)	9
	15c	Describe any proposed additional analyses (such as sensitivity or subgroup analyses, meta-regression)	9
	15d	If quantitative synthesis is not appropriate, describe the type of summary planned	9

Section and topic	Item No	Checklist item	Page
Meta-bias(es)	16	Specify any planned assessment of meta-bias(es) (such as publication bias across studies, selective reporting within studies)	9
Confidence in cumulative evidence	17	Describe how the strength of the body of evidence will be assessed (such as GRADE)	9

For peer review only

BMJ Open

Prevalence of vitamin D and calcium deficiencies and their health impacts on women of childbearing age: a protocol for systematic review and meta-analysis

Journal:	*BMJ Open*
Manuscript ID	bmjopen-2021-049731.R2
Article Type:	Protocol
Date Submitted by the Author:	30-Nov-2021
Complete List of Authors:	Silveira, Erika; Federal University of Goias, Department of Clinical Medicine; University College London, Department of Clinical Medicine<br>Moura, Letícia ; UFG, Department of Clinical Medicine<br>Castro, Maria ; Federal University of Goias, Department of Clinical Medicine<br>Kac, Gilberto; Federal University of Rio de Janeiro<br>Noll, Priscilla; Goiano Federal Institute, Department of Obstetrics and Gynecology; University of São Paulo, Department of Obstetrics and Gynecology<br>de Oliveira, Cesar; University College London, Epidemiology and Public Health<br>Noll, Matias; Instituto Federal Goiano, Public Health; University of Southern Denmark,
**Primary Subject Heading**:	Nutrition and metabolism
Secondary Subject Heading:	Obstetrics and gynaecology
Keywords:	NUTRITION & DIETETICS, EPIDEMIOLOGY, Protocols & guidelines < HEALTH SERVICES ADMINISTRATION & MANAGEMENT

SCHOLARONE™
Manuscripts

Prevalence of vitamin D and calcium deficiencies and their health impacts on women of childbearing age: a protocol for systematic review and meta-analysis

Erika Aparecida Silveira[1,2]

Letícia de Almeida Nogueira e Moura[1]

Maria Clara Rezende Castro[1]

Gilberto Kac[3]

Priscilla Noll[4,5]

Cesar de Oliveira[2]

Matias Noll[1,4,6]

[1]Department of Clinical Medicine, Faculty of Medicine, Health Science Post-graduate Program, Federal University of Goias, Goiás, Brazil

[2]Department of Epidemiology & Public Health, Institute of Epidemiology & Health Care, University College London, London, UK

[3]Federal University of Rio de Janeiro, Rio de Janeiro, Brazil

[4]Goiano Federal Institute, Goiás, Brazil

[5]Department of Obstetrics and Gynecology, Medical School, University of São Paulo, São Paulo, Brazil

[6]Department of Sports Science and Clinical Biomechanics, University of Southern Denmark, Odense, Denmark

Corresponding author: Erika A. Silveira: erikasil@terra.com.br

For peer review only

ABSTRACT

Introduction: No systematic reviews has synthesized data on the available evidence to determine the prevalence of calcium and vitamin D deficiencies as a public health problem globally. Therefore, this study presents a protocol for conducting a review and meta-analysis to estimate the prevalence of calcium and vitamin D serum deficiencies in women of childbearing age and stratify these data by age group, urban and rural area, world region, and pregnant/non-pregnant women whenever possible. **Methods and analysis:** The systematic review protocol involves conducting a literature search in the following databases: PubMed, LILACS, Embase, Scopus, and Web of Science. The selected articles will be checked thoroughly, including the references to include gray literature. Cross-sectional studies and baseline data from cohort studies or clinical and community trials conducted with women of childbearing age with representative probabilistic sampling will be included. Two independent researchers will be responsible for article selection and data extraction, and discrepancies, if any, will be dealt with by a third reviewer. Methodological quality and risk of bias will be analyzed using the Grading of Recommendations, Assessment, Development, and Evaluations, and JBI checklist, respectively. The heterogeneity of the estimates between studies will also be evaluated. Dissemination of the key findings from the systematic review will help identify priorities for action, establish dietary guidelines, develop health-related public policies, and reduce and combat micronutrient deficiencies among women of childbearing age and their children.

Ethics and dissemination: Formal ethical approval is not required, and findings will be published in a peer-reviewed journal.

PROSPERO registration number CRD42020207850.

Keywords: Micronutrients, Calcium, Vitamin D, Childbearing age, Systematic Review Protocol, Nutritional Deficiencies, Epidemiology

For peer review only

Strengths and limitations of this study

► This systematic review intends to estimate the prevalence of calcium and vitamin D serum deficiencies in women of childbearing age and stratify these data by age group, urban and rural area, world region, and pregnant/non-pregnant women whenever possible;

► The following five databases will be included to identify scientific articles: PubMed, LILACS, Embase, Scopus, and Web of Science;

► Meta-analysis will be evaluated using fixed-effects or random-effects methods according to the studies' homogeneity whenever possible;

► Potential heterogeneity of measurements and assessments of micronutrient deficiency in research may be a limitation.

For peer review only

INTRODUCTION

Nutritional deficiencies have multiple etiologies related to factors such as nutrient loss, malabsorption, inadequate intake, and even ignorance of the importance of nutritional care during pregnancy[1]. Women of childbearing age are part of the highly vulnerable population and often experience these deficiencies, especially during pregnancy [2–5]. Besides, maternal nutritional status influences the pregnancy outcomes and child's health and, as it is necessary to supply the needs of both the mother and the fetus[3–5].

Vitamin D deficiency is considered a significant public health problem and affects approximately one billion people worldwide [6,7]. However, there are significant data gaps in measuring this problem magnitude in many countries[8]. The estimated prevalence of vitamin D deficiency (20ng/ml) was around 37% in Canada and 40% in Europe[9]. A systematic review with a meta-analysis of observational studies found a significant association between low vitamin D levels and all-cause mortality [10]. Regarding the effectiveness of vitamin D on specific diseases on this population the evidences still are not enough, and they should only refer to meta-analyses of randomized trials [10–14]. Specifically for pregnant women, systematic reviews with meta-analysis of randomized clinical trials found that vitamin D supplementation can effectively prevent pre-eclampsia [11] and reduce maternal insulin resistance [12]. Furthermore, it is associated with high circulating levels of 25(OH)D, birth weight and length [13], and fetal or neonatal mortality [14]

Vitamin D levels interfere with calcium metabolism[15]. Calcium is a micronutrient that plays an essential role in muscle contraction and regulates water balance in cells[16]. Hypocalcemia symptoms, characterized by low calcium levels, are variable, ranging from mild disorders such as perioral paresthesia to severe life-threatening ones such as cardiac arrhythmias[17]. In pregnant women, this imbalance is characterized by a lower serum concentration and a higher concentration in the cells, thereby causing increased blood pressure in women with pre-eclampsia[18,19].

Previous studies have evaluated the prevalence of vitamin D and calcium deficiencies in women of childbearing age[20–22]. However, at present, there is no systematic review that synthesizes the state of art of this relevant public health problem nationally and globally. Therefore, this study aimed to estimate the prevalence of calcium

For peer review only

and vitamin D serum deficiencies in women of childbearing age. In addition, the data will be stratified by age group, urban and rural areas, geographical location, and pregnant/non-pregnant women through a systematic review followed by a meta-analysis.

METHODS AND ANALYSIS

This systematic review and meta-analysis will be written according to the Preferred Reporting Items for Systematic Reviews and Meta-Analyses Protocols (PRISMA). The protocol for this systematic review was registered on the Prospero platform (CRD42020207850). This review follows the Population, Intervention, Comparison, and Outcome (PICO) structure, with the population (P) women of childbearing age, the intervention (I) no intervention on this study, the comparison (C) being conducted between subgroups in childbearing age as pregnant and not pregnant, and the outcome (O) being calcium and vitamin D deficiencies. Therefore, our systematic review will be conducted based on the following research question: 'What is the prevalence of calcium and vitamin D serum deficiencies in women of childbearing age?'

Search strategy and databases

A systematic review of the literature will be conducted to identify and collect bibliographic data in the following databases: PubMed (National Library of Medicine, United States), LILACS (Latin American and Caribbean Health Sciences Literature), Embase, Scopus, and Web of Science databases (**Figure 1**). The databases will be searched in December 2021 to identify the potential articles to be included in the systematic review The search strategy will be complemented by screening the reference list of the included studies and relevant systematic reviews on the following months.

Different search strategies will include Medical Subject Heading (MeSH) terms and relevant keywords related to vitamin D deficiency and specific terms related to calcium deficiency to ensure that all articles of interest are identified using all the descriptors and Boolean operators of this systematic review protocol (**Supplementary Table**).

Eligibility criteria

For peer review only

Inclusion criteria

- Studies that provide data on the prevalence of calcium and/or vitamin D serum deficiency in women of childbearing age (15-49 years old or menarche and menopause).
- Studies with representative population-based samples in hospitals, health centres, or outpatient clinics.
- Prevalence data on women of different age groups, such as adolescents, pregnant women, lactating women, and premenopausal adult women.
- Studies with cross-sectional design and data from longitudinal studies (cohort studies) or intervention studies such as clinical trials or community trials providing they have prevalence information in a specific point of time. Articles in English, Portuguese, and Spanish language were selected.

Exclusion criteria

- Opinion articles, comments, or editorials.
- Duplicates: The most comprehensive one that reports the largest sample size will be considered for studies published as more than one article.
- Studies with primary data not accessible even after request.
- Case-control studies, narrative reviews, and case series.
- Studies conducted among athlete women of any sports modality.
- Studies conducted among women with specific diseases:
    - *Autoimmune diseases:* lupus, psoriasis, thyroiditis, rheumatoid arthritis, multiple sclerosis
    - *Eating disorders:* Anorexia, Bulimia
    - *Hematological diseases:* Thalassemia, Sickle Cell Disease
    - *Respiratory diseases:* COPD, Asthma, Pneumonia, Respiratory Infections, Tuberculosis
    - *Chronic diseases such as:* heart failure, kidney failure, liver diseases, chronic kidney disease, cardiac diseases, nephrotic syndrome, AIDS, inflammatory bowel disease, hypothyroidism, sepsis, cancer
    - *Genetic diseases and syndromes:* Mutation in the vitamin D receptor, Cystic Fibrosis, Prader-Willi

For peer review only

- *Neurological or psychiatric illnesses:* Epilepsy or use of antiepileptic medication, ADHD, schizophrenia
- *Trauma, post-surgical:* Burns, Post-bariatric, in the treatment of recent fractures or orthopaedic/osteoarticular diseases
- Patients in intensive care, urgency and emergency and palliative care
- Women with hypo and hyperparathyroidism
- Small number of participants (< 50)
- Studies conducted among indigenous women

Reviewers' training

The authors responsible for assessing article eligibility will be trained on using the inclusion and exclusion criteria. Thus, an eligibility test will be conducted on 50 titles and abstracts before codifying the articles. They will also receive training on instruments to assess the risk of bias used on five articles that have not been included. Rayyan and Mendeley software will be used for the selection steps.

Review process

After completing the search strategy, the identified articles will be gathered and imported into Mendeley software. Duplicate articles will be deleted. The articles will be selected by two independent reviewers (LM and MC). The titles will be read in the first stage, and the abstracts in the second stage. Finally, in the third stage, the entire article will be read. Disagreements between reviewers will be assessed by a third reviewer (EAS). Eligibility will be determined according to the inclusion and exclusion criteria. The percentage of agreement and the Kappa coefficient will be calculated to assess reliability between reviewers' assessments. At the end of the process, the selected articles will be included in the systematic review.

Data extraction and risk of bias assessment

Data extraction will be performed using a spreadsheet that considers the following aspects: author/year, type of study, place of study, age group, type of sample/sample size, place of residence (urban/rural), continent/world region (Asia, Europe, Africa, Latin America, North America, and Oceania), pregnancy status (yes/no), lactating (yes/no), micronutrients analyzed, the technique used, cut-off points, and results, i.e., prevalence/impact of calcium or vitamin D deficiencies (**Table 1**).

For peer review only

In this study, we will use the Joanna Briggs Institute development tool of a critical appraisal tool for use in systematic reviews addressing questions of prevalence[23]. This tool has excellent methodological rigor, handling essential items related to the methodological quality of the studies[24]. To ensure the quality of the study, the tool consists of ten questions that assess criteria such as representativeness and sample size, recruitment of participants, and data analysis[23].

The Grading of Recommendations, Assessment, Development, and Evaluations (GRADE) will be used to assess the strength of evidence[25]. The quality of evidence will be classified into four grades: high, moderate, low, or very low[25].

The data will be extracted and evaluated by two independent reviewers (LM and MC). Disagreements will be resolved by a third reviewer (MN). To obtain relevant data not described in the manuscript, a researcher (MC) will contact the article's authors. The authors of the included studies' potential conflicts of interest and ethical information will also be described.

Patient and Public Involvement

No patient involved

Statistical analysis

Meta-analysis will be performed using random-effects methods. Chi-square test with a significance level ($p < 0.05$) will be used to assess heterogeneity, and the I-square statistic (I^2) will be used to determine the magnitude of inconsistency. I^2 results above 75% will indicate a high heterogeneity, those between 25% and 75% will characterize moderate heterogeneity, and those below 25% will indicate low heterogeneity[26–28].

According to the available literature, sensitivity and estimation of the prevalence of vitamin D and calcium deficiencies will be analyzed. The data will be stratified according to region, age, and urban/rural area subgroups. If applicable, meta-regression will be performed to explain the studies' heterogeneity considering $p < 0.05$ for each stratification subgroup. We will estimate the pooled prevalence's by obtaining the following information from each study: the number of women with calcium and vitamin D deficiencies and sample size total. Analyses will be conducted using R through the

For peer review only

package Meta. The results will be presented as prevalence with 95% confidence interval (CI95%).

The primary outcome of the present study is the prevalence of serum calcium and vitamin D deficiencies in women of childbearing age.

The prevalence of circulating 25(OH)D concentrations below certain thresholds, such as the percentage of values <30 nmol/L or <50 nmol/L will be presented. Selected studies will be grouped in descriptive Tables according to their cut-offs. This way, we will highlight and discuss the current controversies and the need for a standard classification of vitamin D status. This will be one of the scientific contributions of our systematic review.

DISCUSSION

Nutritional deficiencies affect pregnant and non-pregnant women, especially those living in poorer regions like the Brazilian northeast [29–33]. Thus, such nutritional deficiencies need to be reviewed nationally and globally as public health problems [34,35]. Therefore, it is essential to estimate the extension of this problem. To the best of our knowledge, there are no systematic reviews and meta-analyses that synthesize data on the available evidence to assess the extent of the prevalence of calcium and vitamin D deficiencies in women of childbearing age.

Obstacles in implementing policies and public health programs to combat nutritional deficiencies can be exacerbated by inequalities in health service provision and access [36]. Therefore, it is essential to gather, organize, and systematize the available data on the prevalence of the main vitamin and mineral deficiencies to develop intervention plans, list action priorities, and improve existing public health policies for women of childbearing age.

This systematic review protocol is important to improve knowledge on global and Brazilian realities on the prevalence of vitamin D and calcium deficiency in this population group. This study is expected to contribute to suggestions for priority action strategies and the development of public health policies to minimize the problem and prevent the occurrence of new cases and assist in establishing intervention protocols to reduce the main deficiencies according to the priorities indicated in the final report. However, some limitations of this research need to be highlighted. First, the potential heterogeneity of measurements and assessments of micronutrient deficiency in research.

For peer review only

Second, studies may have used different methods, making it difficult to conduct a meta-analysis.

Women of childbearing age, especially pregnant women, are part of a highly vulnerable group that develops nutritional deficiencies, and the maternal nutritional status influences gestational outcomes and the child's health. Considering the above, assessing the prevalence of vitamin D and calcium deficiencies could help identify knowledge gaps on the magnitude of these deficiencies, warranting further studies. From this perspective, disseminating the results of this systematic review protocol will be helpful and vital to encourage research and identifying priorities, establishing dietary guidelines, and reducing and combating micronutrient deficiencies in this population.

Ethics statements

Patient consent for publication

Not required. No patient was involved.

Ethics and Dissemination

A systematic review does not require ethical approval since it synthesizes publicly available literature and will not include individual patient data. The results of the scoping review will be submitted for publication to a scientific journal and presented at relevant conferences.

Acknowledgment

The authors thank CNPq and IF Goiano for providing partial support.

Funding sources/sponsors

This study received support from the This study received support from the MS-SCTIE-Decit/*CNPq* Nº 442932/2019-7. Dr Cesar de Oliveira is supported by the Economic and Social Research Council (Grant ES/T008822/1)

Conflicts of interest

For peer review only

The authors of this systematic review and meta-analysis declare no conflicts of interest. This review will analyze whether the studies report a possible conflict of interest and approval by the Research Ethics Committee (ELIA et al., 2016). It will also verify if the studies followed the ethical requirements recommended in the Declaration of Helsinki, respecting all the ethical principles established in resolution 466/2012 of the National Health Council (BRASIL, 2012) or the norms and ethical resolutions in force at the time of data collection.

Contributorship statement

Conceptualization, ES, LA, MC, and MN; methodology, ES, LA, MC, and MN; formal analysis, ES, LA, MC, and MN; investigation, ES, LA, MC, GK, PN, CO, and MN; resources, ES.; writing—original draft preparation, ES, LA, MC, GK, PN, CO, and MN; writing—review and editing, ES, LA, MC, GK, PN, CO, and MN; visualization ES, LA, MC, GK, PN, CO, and MN; supervision, MN, and ES.; funding acquisition, ES. All authors have read and agreed to the published version of the manuscript.

REFERENCES

1 Miranda VIA, da Silva Dal Pizzol T, Silveira MPT, *et al.* The use of folic acid, iron salts and other vitamins by pregnant women in the 2015 Pelotas birth cohort: is there socioeconomic inequality? *BMC Public Health* 2019;**19**:889. doi:10.1186/s12889-019-7269-0

2 Barger MK. Maternal Nutrition and Perinatal Outcomes. *Journal of Midwifery & Women's Health* 2010;**55**:502–11. doi:10.1016/j.jmwh.2010.02.017

3 Iqbal S, Ekmekcioglu C. Maternal and neonatal outcomes related to iron supplementation or iron status: a summary of meta-analyses. *The Journal of Maternal-Fetal & Neonatal Medicine* 2019;**32**:1528–40. doi:10.1080/14767058.2017.1406915

4 Obeid R, Murphy M, Solé-Navais P, *et al.* Cobalamin Status from Pregnancy to Early Childhood: Lessons from Global Experience. *Advances in Nutrition: An International Review Journal* 2017;**8**:971–9. doi:10.3945/an.117.015628

5 Nauman, Nuzhat ; Jalali, Samina; Shami, Sajjad ; Rafiq, Shireen; Große, Greta; Hilger, Alina C .; Zhang, Rhong; Mansoor, Saira; Ludwig, Michael L.; Reutter H. Low maternal folate concentrations and maternal MTHFR C677T polymorphism are associated with an increased risk for neural tube defects in offspring: a case-control study among Pakistani

For peer review only

case and control mothers. *Asia Pac J Clin Nutr* 2018;**27**:253–60. doi:10.6133/apjcn.032017.10

6 Mithal A, Wahl DA, Bonjour J-P, *et al.* Global vitamin D status and determinants of hypovitaminosis D. *Osteoporosis International* 2009;**20**:1807–20. doi:10.1007/s00198-009-0954-6

7 Martins MEP, Esmeraldo CUP, Sabiá JPD, *et al.* Vitamin D Postpartum Concentrations: Relationship with Nutritional Condition and Morbidities during Pregnancy. *Journal of Pregnancy* 2018;**2018**:1–6. doi:10.1155/2018/1070528

8 Roth DE, Abrams SA, Aloia J, *et al.* Global prevalence and disease burden of vitamin D deficiency: a roadmap for action in low- and middle-income countries. *Annals of the New York Academy of Sciences* 2018;**1430**:44–79. doi:10.1111/nyas.13968

9 Amrein K, Scherkl M, Hoffmann M, *et al.* Vitamin D deficiency 2.0: an update on the current status worldwide. *European Journal of Clinical Nutrition* 2020;**74**:1498–513. doi:10.1038/s41430-020-0558-y

10 Garland CF, Kim JJ, Mohr SB, *et al.* Meta-analysis of All-Cause Mortality According to Serum 25-Hydroxyvitamin D. *American Journal of Public Health* 2014;**104**:e43–50. doi:10.2105/AJPH.2014.302034

11 Fogacci S, Fogacci F, Banach M, *et al.* Vitamin D supplementation and incident preeclampsia: A systematic review and meta-analysis of randomized clinical trials. *Clinical Nutrition* 2020;**39**:1742–52. doi:10.1016/j.clnu.2019.08.015

12 Gallo S, McDermid JM, Al-Nimr RI, *et al.* Vitamin D Supplementation during Pregnancy: An Evidence Analysis Center Systematic Review and Meta-Analysis. *Journal of the Academy of Nutrition and Dietetics* 2020;**120**:898-924.e4. doi:10.1016/j.jand.2019.07.002

13 Pérez-López FR, Pasupuleti V, Mezones-Holguin E, *et al.* Effect of vitamin D supplementation during pregnancy on maternal and neonatal outcomes: a systematic review and meta-analysis of randomized controlled trials. *Fertility and Sterility* 2015;**103**:1278-1288.e4. doi:10.1016/j.fertnstert.2015.02.019

14 Bi WG, Nuyt AM, Weiler H, *et al.* Association Between Vitamin D Supplementation During Pregnancy and Offspring Growth, Morbidity, and Mortality. *JAMA Pediatrics* 2018;**172**:635. doi:10.1001/jamapediatrics.2018.0302

15 Fleet JC. The role of vitamin D in the endocrinology controlling calcium homeostasis. *Molecular and Cellular Endocrinology* 2017;**453**:36–45. doi:10.1016/j.mce.2017.04.008

16 Sweeney HL, Hammers DW. Muscle Contraction. *Cold Spring Harbor Perspectives in Biology* 2018;**10**:a023200. doi:10.1101/cshperspect.a023200

17 Bilezikian JP, Brandi ML, Cusano NE, *et al.* Management of Hypoparathyroidism: Present and Future. *The Journal of Clinical Endocrinology & Metabolism* 2016;**101**:2313–24. doi:10.1210/jc.2015-3910

18 Kumar N, Singh AK. Maternal serum uric acid and calcium as predictors of hypertensive disorder of pregnancy: A case control study. *Taiwanese Journal of Obstetrics and Gynecology* 2019;**58**:244–50. doi:10.1016/j.tjog.2019.01.014

19 Abbasalizadeh S, Abam F, Mirghafourvand M, *et al.* Comparing levels of vitamin D, calcium and phosphorus in normotensive pregnant women and pregnant women with

preeclampsia. *Journal of Obstetrics and Gynaecology* 2020;**40**:1069–73. doi:10.1080/01443615.2019.1678575

20 Zhu T, Liu T-J, Ge X, *et al.* High prevalence of maternal vitamin D deficiency in preterm births in northeast China, Shenyang. *International journal of clinical and experimental pathology* 2015;**8**:1459–65.http://www.ncbi.nlm.nih.gov/pubmed/4396334

21 Zosky GR, Hart PH, Whitehouse AJO, *et al.* Vitamin D Deficiency at 16 to 20 Weeks' Gestation Is Associated with Impaired Lung Function and Asthma at 6 Years of Age. *Annals of the American Thoracic Society* 2014;**11**:571–7. doi:10.1513/AnnalsATS.201312-423OC

22 Wang J, Yang F, Mao M, *et al.* High prevalence of vitamin D and calcium deficiency among pregnant women and their newborns in Chengdu, China. *World Journal of Pediatrics* 2010;**6**:265–7. doi:10.1007/s12519-010-0224-x

23 Munn Z, Moola S, Riitano D, *et al.* The development of a critical appraisal tool for use in systematic reviews addressing questions of prevalence. *International Journal of Health Policy and Management* 2014;**3**:123–8. doi:10.15171/ijhpm.2014.71

24 Migliavaca CB, Stein C, Colpani V, *et al.* Quality assessment of prevalence studies: a systematic review. *Journal of Clinical Epidemiology* 2020;**127**:59–68. doi:10.1016/j.jclinepi.2020.06.039

25 Guyatt GH, Oxman AD, Sultan S, *et al.* GRADE guidelines: 9. Rating up the quality of evidence. *Journal of Clinical Epidemiology* 2011;**64**:1311–6. doi:10.1016/j.jclinepi.2011.06.004

26 Rodrigues, Caroline Legramanti; Ziegelman PKlarmann. META-ANALYSIS: A PRACTICAL GUIDE. *Rev HCPA* 2010;**30**:436–47.

27 Higgins JPT. Measuring inconsistency in meta-analyses. *BMJ* 2003;**327**:557–60. doi:10.1136/bmj.327.7414.557

28 Brasil. Ministério da Saúde. Diretrizes metodológicas : elaboração de revisão sistemática e metanálise de ensaios clínicos randomizados. 2012.

29 Saúde. BrasilM da. Pesquisa nacional de demografia e saúde da criança e da mulher (PNDS 2006): Dimensões do processo reprodutivo e da saúde da criança. 2009.

30 Lício JSA, Fávaro TR, Chaves CRM de M. Anemia em crianças e mulheres indígenas no Brasil: revisão sistemática. *Ciência & Saúde Coletiva* 2016;**21**:2571–81. doi:10.1590/1413-81232015218.00532015

31 Achebe MM, Gafter-Gvili A. How I treat anemia in pregnancy: iron, cobalamin, and folate. *Blood* 2017;**129**:940–9. doi:10.1182/blood-2016-08-672246

32 Bezerra AGN, Leal VS, Lira PIC de, *et al.* Anemia e fatores associados em mulheres de idade reprodutiva de um município do Nordeste brasileiro. *Revista Brasileira de Epidemiologia* 2018;**21**. doi:10.1590/1980-549720180001

33 Bastos Maia S, Costa Caminha M, Lins da Silva S, *et al.* The Prevalence of Vitamin A Deficiency and Associated Factors in Pregnant Women Receiving Prenatal Care at a Reference Maternity Hospital in Northeastern Brazil. *Nutrients* 2018;**10**:1271. doi:10.3390/nu10091271

For peer review only

34 Miller R, Spiro A, Stanner S. Micronutrient status and intake in the UK - where might we be in 10 years' time? *Nutrition Bulletin* 2016;**41**:14–41. doi:10.1111/nbu.12187

35 Harding KL, Aguayo VM, Webb P. Hidden hunger in South Asia: a review of recent trends and persistent challenges. *Public Health Nutrition* 2018;**21**:785–95. doi:10.1017/S1368980017003202

36 Kiely ME, Wagner CL, Roth DE. Vitamin D in pregnancy: Where we are and where we should go. *The Journal of Steroid Biochemistry and Molecular Biology* 2020;**201**:105669. doi:10.1016/j.jsbmb.2020.105669

For peer review only

Table 1. Data extraction worksheet.

Author/ year	Study type	Study location	Age group	Sample type/sample size	Place of residence (rural/urban)	Continent/ World region	Pregnant women (yes/no)	Lactating women (yes/no)	Micronutrients analyzed		Technique used	Cutoff points	Prevalence		Notes
									Calcium	Vit. D			Calcium	Vit. D	

For peer review only

Figure 1. PRISMA flow diagram.

For peer review only

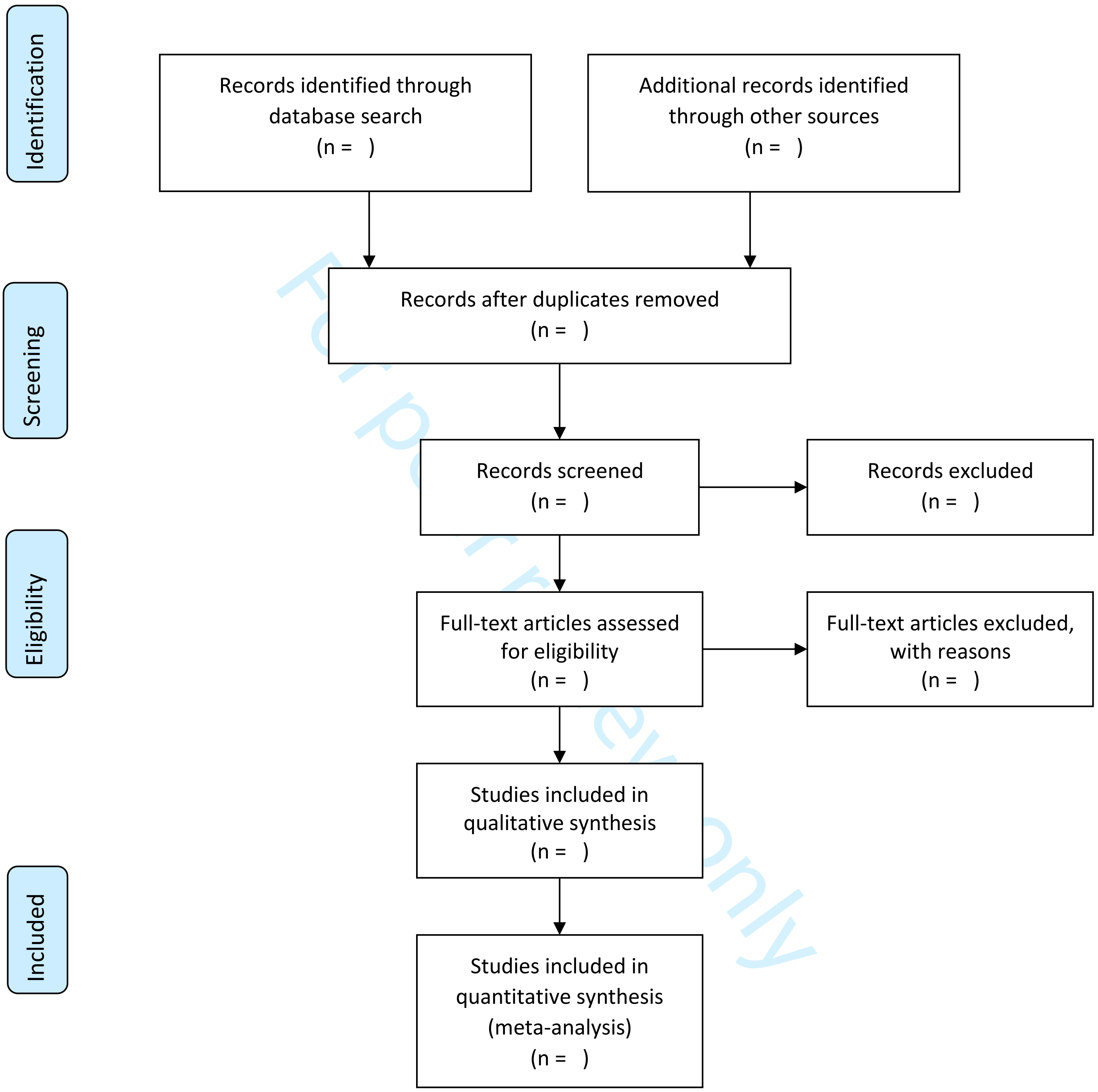
Identification
Screening
Eligibility
Included
Records identified through database search (n =)
Additional records identified through other sources (n =)
Records after duplicates removed (n =)
Records screened (n =)
Records excluded (n =)
Full-text articles assessed for eligibility (n =)
Full-text articles excluded, with reasons (n =)
Studies included in qualitative synthesis (n =)
Studies included in quantitative synthesis (meta-analysis) (n =)

Supplementary Table. Search terms selected according to specific nomenclature for each database

**MEDLINE**	(("Micronutrients" [Title/Abstract] OR "Micronutrients" [MeSH Terms] OR "Deficiency Diseases" [Title/Abstract] OR "Deficiency Diseases" [MeSH Terms] OR "Avitaminosis" [Title/Abstract] OR "Avitaminosis" [MeSH Terms] OR "Deficiencies vitamin" [Title/Abstract] OR "Deficiencies vitamin" [MeSH Terms] OR "Deficiency vitamin" [Title/Abstract] OR "Vitamin deficiency" [Title/Abstract] OR "Vitamin Deficiencies" [Title/Abstract] OR "Serum Levels" [Title/Abstract] OR "Nutritional Status" [Title/Abstract] OR "Nutritional Status" [MeSH Terms] OR "Reduced Level" [Title/Abstract] OR "Reduced Level" [MeSH Terms]) AND ("Women" [Title/Abstract] OR "Women" [MeSH Terms] OR "Girl" [Title/Abstract] OR "Girls" [Title/Abstract] OR "Woman" [Title/Abstract] OR "Adolescent" [Title/Abstract] OR "Adolescent" [MeSH Terms] OR "Teen" [Title/Abstract] OR "Teens" [Title/Abstract] OR "Teenager" [Title/Abstract] OR "Pregnancy" [Title/Abstract] OR "Pregnancy" [MeSH Terms] OR "Gestation" [Title/Abstract] OR "Pregnancies" [Title/Abstract])) AND ("Vitamin D" [Title/Abstract] OR "Vitamin D" [MeSH Term] OR "Vitamin D Deficiency" [Title/Abstract] OR "Vitamin D Deficiency" [MeSH Term] OR "deficiencies vitamin d" [Title/Abstract] OR "Vitamin D Deficiencies" [Title/Abstract] OR "Vitamin D Deficiencies" [MeSH Term] OR "Calcium" [Title/Abstract] OR "Calcium" [MeSH Term] OR "Hypocalcemia" [Title/Abstract] OR "Hypocalcemia" [MeSH Term] OR "Hypocalcemias" [Title/Abstract])
**Embase**	('micronutrients':ab,ti OR 'deficiency diseases':ab,ti OR 'avitaminosis':ab,ti OR 'avitaminoses':ab,ti OR 'deficiencies vitamin':ab,ti OR 'deficiency vitamin':ab,ti OR 'vitamin deficiency':ab,ti OR 'vitamin deficiencies':ab,ti OR 'serum levels':ab,ti OR 'nutritional status':ab,ti OR 'reduced level':ab,ti) AND ('women':ab,ti OR 'girl':ab,ti OR 'girls':ab,ti OR 'woman':ab,ti OR 'adolescent':ab,ti OR 'teen':ab,ti OR 'teens':ab,ti OR 'teenager':ab,ti OR 'pregnancy':ab,ti OR 'gestation':ab,ti OR 'pregnancies':ab,ti) AND ('vitamin d':ab,ti OR 'vitamin d deficiency':ab,ti OR 'deficiencies vitamin d':ab,ti OR 'vitamin d deficiencies':ab,ti OR 'calcium':ab,ti OR 'hypocalcemia':ab,ti OR 'hypocalcemias':ab,ti)
**Scopus**	(TITLE-ABS ("Micronutrients" OR "Deficiency Diseases" OR "Avitaminosis" OR "Avitaminoses" OR "Deficiencies, Vitamin" OR "Deficiency Vitamin" OR "Vitamin Deficiency" OR "Vitamin Deficiencies" OR "Serum Levels" OR "Nutritional Status" OR "Reduced Level")) AND (TITLE-ABS ("Women" OR "Girl" OR "Girls" OR "Woman" OR "Adolescent" OR "Teen" OR "Teens" OR "Teenager" OR "Pregnancy" OR "Gestation" OR "Pregnancies")) AND (TITLE-ABS ("Vitamin D" OR "Vitamin D Deficiency" OR "Deficiencies, Vitamin D" OR "Vitamin D Deficiencies" OR "Calcium" OR "Hypocalcemia" OR "Hypocalcemias"))
**Web of Science**	(TI= "Micronutrients" OR AB= "Micronutrients" OR TI="Deficiency Diseases" OR AB= "Deficiency Diseases" OR TI= "Avitaminosis" OR AB= "Avitaminosis" OR TI= "Avitaminoses" OR AB= "Avitaminoses" OR TI= "deficiencies vitamin" OR AB= "deficiencies vitamin" OR TI= "Deficiency Vitamin" OR AB= "Deficiency Vitamin" OR TI= "Vitamin Deficiency" OR AB="Vitamin Deficiency" OR TI= "Vitamin Deficiencies" OR AB="Vitamin Deficiencies" OR TI= "Serum Levels" OR AB= "Serum Levels" OR TI="Nutritional Status" OR AB="Nutritional Status" OR TI="Reduced Level" OR AB="Reduced Level") AND (TI="Women" OR AB= "Women" OR TI="Girl" OR AB="Girl" OR TI="Girls" OR AB="Girls"

	OR TI="Woman" OR AB="Woman" OR TI="Adolescent" OR AB= "Adolescent" OR TI="Teen" OR AB="Teen" OR TI="Teens" OR AB="Teens" OR TI="Teenager" OR AB="Teenager" OR TI="Pregnancy" OR AB="Pregnancy" OR TI="Gestation" OR AB="Gestation" OR TI="Pregnancies" OR AB="Pregnancies") AND (TI="Vitamin D" OR AB="Vitamin D" OR TI="Vitamin D Deficiency" OR AB= "Vitamin D Deficiency" OR TI="deficiencies vitamin d" OR AB="deficiencies vitamin d" OR TI="Vitamin D Deficiencies" OR AB="Vitamin D Deficiencies" OR TI="Calcium" OR AB="Calcium" OR TI="Hypocalcemia" OR AB= "Hypocalcemia" OR TI="Hypocalcemias" OR AB= "Hypocalcemias")
**LILACS**	(tw:((tw:("Micronutrients")) OR (tw:("Deficiency Diseases")) OR (tw:("Avitaminosis")) OR (tw:("Avitaminoses")) OR (tw:("deficiencies vitamin")) OR (tw:("Deficiency Vitamin")) OR (tw:("Vitamin Deficiency")) OR (tw:("Vitamin Deficiencies")) OR (tw:("Serum Levels")) OR (tw:("Nutritional Status")) OR (tw:("Reduced Level")))) AND (tw:(Women OR Girl OR Girls OR Woman OR Adolescent OR Teen OR Teens OR Teenager OR Pregnancy OR Gestation OR Pregnancies)) AND (tw:(("Vitamin D") OR ("Vitamin D Deficiency") OR ("deficiencies vitamin d") OR ("Vitamin D Deficiencies") OR "Calcium" OR "Hypocalcemia" OR "Hypocalcemias"))

For peer review only

Table
PRISMA-P (preferred reporting items for systematic review and meta-analysis protocols) 2015 checklist: recommended items to address in a systematic review protocol

Section and topic	Item No	Checklist item	Page
**Administrative information**			
Identification	1a	Identify the report as a protocol of a systematic review	1
Update	1b	If the protocol is for an update of a previous systematic review, identify as such	NA
Registration	2	If registered, provide the name of the registry (such as PROSPERO) and registration number	2
Authors:			
Contact	3a	Provide name, institutional affiliation, e-mail address of all protocol authors; provide physical mailing address of corresponding author	1
Contributions	3b	Describe contributions of protocol authors and identify the guarantor of the review	1
Amendments	4	If the protocol represents an amendment of a previously completed or published protocol, identify as such and list changes; otherwise, state plan for documenting important protocol amendments	NA
Support:			
Sources	5a	Indicate sources of financial or other support for the review	11
Sponsor	5b	Provide name for the review funder and/or sponsor	11
Role of sponsor or funder	5c	Describe roles of funder(s), sponsor(s), and/or institution(s), if any, in developing the protocol	11
**Introduction**			

peer review only

Section and topic	Item No	Checklist item	Page
Rationale	6	Describe the rationale for the review in the context of what is already known	4
Objectives	7	Provide an explicit statement of the question(s) the review will address with reference to participants, interventions, comparators, and outcomes (PICO)	4/5
**Methods**			
Eligibility criteria	8	Specify the study characteristics (such as PICO, study design, setting, time frame) and report characteristics (such as years considered, language, publication status) to be used as criteria for eligibility for the review	5
Information sources	9	Describe all intended information sources (such as electronic databases, contact with study authors, trial registers or other grey literature sources) with planned dates of coverage	5
Search strategy	10	Present draft of search strategy to be used for at least one electronic database, including planned limits, such that it could be repeated	5/6/7
Study records:			
Data management	11a	Describe the mechanism(s) that will be used to manage records and data throughout the review	8
Selection process	11b	State the process that will be used for selecting studies (such as two independent reviewers) through each phase of the review (that is, screening, eligibility and inclusion in meta-analysis)	8

Section and topic	Item No	Checklist item	Page
Data collection process	11c	Describe planned method of extracting data from reports (such as piloting forms, done independently, in duplicate), any processes for obtaining and confirming data from investigators	8
Data items	12	List and define all variables for which data will be sought (such as PICO items, funding sources), any pre-planned data assumptions and simplifications	8
Outcomes and prioritization	13	List and define all outcomes for which data will be sought, including prioritization of main and additional outcomes, with rationale	8
Risk of bias in individual studies	14	Describe anticipated methods for assessing risk of bias of individual studies, including whether this will be done at the outcome or study level, or both; state how this information will be used in data synthesis	8/9
Data synthesis	15a	Describe criteria under which study data will be quantitatively synthesised	9
	15b	If data are appropriate for quantitative synthesis, describe planned summary measures, methods of handling data and methods of combining data from studies, including any planned exploration of consistency (such as I^2, Kendall's τ)	9
	15c	Describe any proposed additional analyses (such as sensitivity or subgroup analyses, meta-regression)	9
	15d	If quantitative synthesis is not appropriate, describe the type of summary planned	9

Section and topic	Item No	Checklist item	Page
Meta-bias(es)	16	Specify any planned assessment of meta-bias(es) (such as publication bias across studies, selective reporting within studies)	9
Confidence in cumulative evidence	17	Describe how the strength of the body of evidence will be assessed (such as GRADE)	9

For peer review only
